# A holistic approach to dissecting SPARC family protein complexity reveals FSTL-1 as an inhibitor of pancreatic cancer cell growth

**DOI:** 10.1038/srep37839

**Published:** 2016-11-25

**Authors:** Katrina Viloria, Amanda Munasinghe, Sharan Asher, Roberto Bogyere, Lucy Jones, Natasha J. Hill

**Affiliations:** 1 Department of Biomolecular Sciences, Kingston University, Kingston-upon-Thames, UK

## Abstract

SPARC is a matricellular protein that is involved in both pancreatic cancer and diabetes. It belongs to a wider family of proteins that share structural and functional similarities. Relatively little is known about this extended family, but evidence of regulatory interactions suggests the importance of a holistic approach to their study. We show that Hevin, SPOCKs, and SMOCs are strongly expressed within islets, ducts, and blood vessels, suggesting important roles for these proteins in the normal pancreas, while FSTL-1 expression is localised to the stromal compartment reminiscent of SPARC. In direct contrast to SPARC, however, FSTL-1 expression is reduced in pancreatic cancer. Consistent with this, FSTL-1 inhibited pancreatic cancer cell proliferation. The complexity of SPARC family proteins is further revealed by the detection of multiple cell-type specific isoforms that arise due to a combination of post-translational modification and alternative splicing. Identification of splice variants lacking a signal peptide suggests the existence of novel intracellular isoforms. This study underlines the importance of addressing the complexity of the SPARC family and provides a new framework to explain their controversial and contradictory effects. We also demonstrate for the first time that FSTL-1 suppresses pancreatic cancer cell growth.

The extracellular matrix (ECM) provides both structural support and regulates cellular responses. Diabetes results from an insufficiency of insulin-producing islet β cells and a failure of compensatory β cell growth and regeneration^1^. Worldwide, diabetes affects 415 million people and this figure is estimated to increase to 642 million by 2040^2^. The discovery of therapeutic mechanisms to stimulate β cell growth would allow physiological control of glucose levels and avoid many of the side effects and risks associated with poorly managed disease^1^. The pancreas is also the site of one of the most lethal types of cancer. Pancreatic ductal adenocarcinoma (PDAC) arises in the ductal epithelial cells of the pancreas and has one of the lowest 5-year survival rates of all cancers (<5%). It also ranks as the seventh most common cause of death from cancer worldwide^3^. An underlying feature common to both diseases is the dysregulation of cell growth and survival, in which the extracellular matrix is likely to play an important regulatory role.

Pancreatic islets are surrounded by a basement membrane composed of ECM proteins such as collagens, laminin, and fibronectin^4^,^5^,^6^. Islets cultured in a collagen matrix showed improved β cell mass, survival and glucose stimulated-insulin secretion compared to islets cultured in 2D^7^,^8^. The ECM and associated integrin signalling have also been implicated in the pathogenesis of diabetes. For example, mice deficient in β1 integrin have decreased β cell mass, proliferation, glucose tolerance and insulin production^9^,^10^.

In pancreatic cancer, the production of collagens I, III, and fibronectin is increased^11^,^12^,^13^ and matrix remodelling enzymes such as MMPs and their inhibitors contribute to pancreatic cancer progression and metastasis^14^,^15^. Disruption of the basement membrane composition can lead to changes in apicobasal polarity and cause changes in cell shape and behavior. This has been shown to drive increased cell proliferation and tumourigenesis^16^,^17^,^18^. Stromal cells such as stellate cells, fibroblasts, endothelial cells and macrophages produce ECM proteins and also growth factors and cytokines that make up the extracellular environment. One of the hallmarks of PDAC is an extensive stroma that creates a protective capsule around the tumour and can constitute up to 80% of the tumour mass^19^. Indeed, the tumour-stroma itself is now considered a therapeutic target in pancreatic cancer^20^,^21^. The regulation of cell growth and migration by the ECM and stromal cells underlies their important role in the progression of both pancreatic cancer and diabetes.

Central to the regulation of ECM structure and cell-matrix interactions are non-structural matricellular proteins such as the SPARC family^22^. SPARC, or secreted protein acidic and rich in cysteine, is produced by stromal cells and regulates matrix remodelling and cell-matrix interactions^23^. SPARC has been shown to regulate cell adhesion and there is also evidence for a role in the regulation of cell growth and proliferation. SPARC modulates cell growth responses to a range of growth factors including TGF-β^24^,^25^,^26^,^27^,^28^,^29^,^30^, binds to both β1 integrins and collagen, and regulates collagen assembly and fibrillogenesis^31^,^32^,^33^. Hence SPARC determines cell responses to the ECM and controls multiple pathways fundamental to cell growth and adhesion.

SPARC is known to be highly expressed during development, wound healing and tissue regeneration and to play a role in angiogenesis, tumourigenesis and fibrosis^23^,^34^,^35^. In pancreatic cancer, stromal SPARC over-expression is strongly associated with poor patient prognosis^36^,^37^. Insulin resistance, diabetes and obesity are also associated with elevated levels of SPARC^38^,^39^,^40^. We have previously shown that SPARC is expressed by pancreatic stromal cells and is localised to the islet basement membrane. SPARC inhibits β cell and islet responses to growth factors^28^ and can also influence β cell function^41^. The production of SPARC by pancreatic stellate cells is regulated by metabolic parameters suggesting that SPARC may influence β cell loss and dysfunction in patients with type 2 diabetes^28^. SPARC is therefore involved in a number of pancreatic diseases, and SPARC expression and function in the pancreas is relatively well characterised. However, very little is known about the wider SPARC family of proteins, despite structural and functional similarities that suggest potentially similar roles.

The wider SPARC family consists of seven additional proteins: SPARC-like 1 or Hevin, SPOCK-1, -2, & -3, SMOC-1 and -2, and FSTL-1. As shown in Fig. 1, these proteins share three main domains: domain I – a highly acidic region with low affinity calcium binding; domain II- a follistatin-like domain consisting of kazal-like domains involved in growth factor binding; and domain III- a high affinity calcium binding domain with 2 EF hands [also known as the EC domain] that is involved in collagen interactions. The EC and follistatin-like domains are well conserved within the family. In contrast, domain I is highly variable and, other than retaining an overall acidic nature, can be regarded as distinct in each SPARC family protein. In addition to these three domains, thyroglobulin domains are present in the SPOCK and SMOC proteins, a glycosaminoglycan-binding domain is present in the SPOCK proteins, and FSTL-1 contains a von Willebrand factor type-C domain.

Hevin is structurally the closest relative of SPARC and the two proteins have many overlapping roles. Like SPARC, Hevin can be anti-adhesive, modulates cell shape, binds to collagen and regulates collagen assembly^42^,^43^,^44^,^45^. While the role of Hevin in diabetes has not yet been examined, Hevin has been implicated in many cancers. Similar to SPARC, Hevin mRNA is overexpressed in pancreatic cancer compared to normal tissue. In fact, it has been suggested that proteolytic cleavage of Hevin creates a ‘SPARC-like fragment’ that can functionally compensate for SPARC, and that as such Hevin acts as a SPARC reservoir^46^,^47^.

The SPOCK proteins also influence matrix characteristics, primarily through MMP regulation. For example, SPOCK-1 and SPOCK-3 inhibit membrane-type MMP (MT-MMP) activation of pro-MMP2^48^, while SPOCK-2 abrogates the inhibition of MT-MMPs by SPOCK-1 and SPOCK-3^49^. MMPs are important regulators of cell migration and cell proliferation, for example through TGF-β activation^50^. The role of SPOCKs in diabetes is unknown but, as for SPARC, high expression of SPOCK-1 in the desmoplastic stroma of PDAC has been associated with poor prognosis^51^. In other cancers, the increase in SPOCK-1 expression by TGF-β is thought to be involved in epithelial-mesenchymal transition (EMT)^52^, and tumour invasion of glioma cells was inhibited by SPOCK-3 transfection^49^. These studies suggest a role for SPOCK proteins in matrix remodelling and metastasis, and emphasize the importance of co-regulation between the SPOCK subfamily of proteins.

Although the role of SMOC proteins in the pancreas has not yet been examined, SMOC-1 is known to be an antagonist for TGF-β superfamily-BMP proteins^53^ and is important for stem cell differentiation^54^. SMOC-1 may also influence tissue remodelling by interaction with the matricellular protein tenascin-C^55^. In breast cancer, the SMOC-1 gene was found to be hypermethylated^56^ but otherwise very little is known about the role of SMOC-1 in human disease. There is similarly little known about the role of SMOC-2, although there is evidence that SMOC-2 has mitogenic properties and is an important regulator of proliferation^57^, and can synergise with VEGF and bFGF-induced angiogenesis in endothelial cells^58^. Like SPARC, SMOC-2 may therefore regulate growth factor signalling. As for many matricellular proteins, SMOC-2 can also bind to integrins, and can promote cell attachment and focal adhesion formation^59^.

FSTL-1 has the lowest homology to SPARC. It was first discovered as a TGF-β-induced protein and it has also been shown to bind directly to the TGF-β superfamily including BMPs^60^,^61^. In bone cancer cells, FSTL-1 was shown to promote metastasis^62^. On the other hand, the opposite was observed in ovarian and endometrial cancers where FSTL-1 was reported to be a tumour suppressor and increased the expression of apoptotic molecules such as caspases, and inhibited MMP2 expression and cell invasion^63^. As yet, the role of FSTL-1 in pancreatic cancer and diabetes is unknown. However, FSTL-1 is closely related to other follistatin-like proteins that have been implicated in pancreatic diseases. For example, FSTL-2, also known as IGFBP-7, is associated with insulin resistance and type II diabetes^64^,^65^, and FSTL-2 down-regulation is associated with poor prognosis in PDAC patients^66^. Furthermore, FSTL-3 knockout mice have increased pancreatic islet number and improved insulin sensitivity^67^.

Hence, there is evidence that the extended SPARC family of proteins play similar roles to SPARC in regulating both the matrix and cellular responses to their environment. There is also growing evidence of coordinated regulation between SPARC family members, for example interactions between SPOCK proteins that regulate their function, and the potential overlapping function of Hevin and SPARC^68^. The presence of active proteolytic fragments further adds to this complexity, and the SPARC family of proteins can also interact with other matricellular proteins. For example, SPARC interacts with TSP-1^69^, and SMOC-1 with tenascin-C^55^. Ultimately, it will therefore be necessary to dissect the complexity and interactions of the SPARC family of matricellular proteins, and this may help resolve some of the contradictions and controversy regarding their function. The urgency is enhanced by the clinical importance of SPARC and other matricellular proteins as potential biomarkers and therapeutic targets^70^. To achieve this it will be necessary to take a systematic approach to understanding the function and complexity of the SPARC family of proteins. The aim of this study was therefore to investigate the expression of the extended SPARC family in the pancreas and provide a holistic analysis addressing their complexity in order to understand their role in pancreatic diseases.

## Results and Discussion

### Expression of the SPARC family of proteins in the pancreas

We have previously defined the expression of SPARC in the pancreas^28^, but the expression of other members of the SPARC family is currently not known. We therefore used immunohistochemistry to analyse the expression of all members of the wider SPARC family of proteins in pancreas sections, examining expression specifically in islets, ducts and blood vessels.

Hevin was detected throughout the islets, with stronger staining in selected cells primarily at the islet periphery, as shown by the solid arrows in Fig. 2a panels (i) & (ii). In these cells, Hevin staining was observed primarily in the cytoplasm, although nuclear staining is apparent in some islet cells. Staining of the islet basement membrane was also observed, as indicated by the dotted arrows in (i). Hevin was also expressed in blood vessels (iii), in connective tissues (iv), in ductal cells (v), and in selected cells in the acinar tissue (vi). Hevin expression in the normal mouse pancreas is therefore distinct to that observed in the human pancreas, where expression is much more restricted, and in islets appears localised specifically to stromal cells^71^, as we have observed previously for SPARC^28^.

As shown in Figs 2 and 3, the SPOCKs are also detected throughout islets, with strikingly strong expression of SPOCK-1 and SPOCK-3 in particular. The SPOCKs are also expressed in blood vessels, ductal cells and ductal basement membranes, and in selected acinar cells. SPOCK-1 staining was observed in the cytoplasm (Fig. 2b i–iii) but could also clearly be observed at the cell surface or extracellularly in ducts and in selected islet cells (arrows in Fig. 2b i and v), and was largely absent in the nucleus. A distinct perinuclear staining was observed in selected acinar cells (arrow in Fig. 2b vi). Similar to Hevin, SPOCK-2 also showed higher expression in cells at the islet periphery, and again staining was primarily evident in the cytoplasm as shown in Fig. 2c(i & ii). SPOCK-3 was highly expressed in all islet cells and with primarily a cytoplasmic staining pattern (Fig. 3a i–iii), although a more restricted perinuclear staining was observed in scattered cells throughout the acinar tissue (Fig. 3a vi). The high levels of SPOCK expression in islets suggests that these proteins may play an important role in normal islet function, and it will be important for future studies to examine this further.

Both SMOC-1 (Fig. 3b) and SMOC-2 (Fig. 3c) were detected throughout the islet, with a range of staining intensity in different cells. For both proteins, the staining was again largely cytoplasmic, though strong nuclear SMOC-1 staining could also be observed in selected cells (eg arrow in Fig. 3b iii). The SMOCs were also expressed in blood vessels, in ductal cells (Fig. 3b and c panels iv, v & solid arrows in vi), as well as in the surrounding connective tissue (dotted arrows in vi).

Within islets, FSTL-1 was expressed primarily in blood vessels and the islet basement membrane, although some weak diffuse staining can be observed throughout the islet (Fig. 4 i & ii). FSTL-1 was also expressed in large and small blood vessels throughout the endocrine and exocrine pancreas as well as in connective tissue (iii & iv). In ducts, FSTL-1 was clearly detected at the cell surface (vi), although some staining was also observed in the cytoplasm of ductal cells (v).

**Figure 1:**
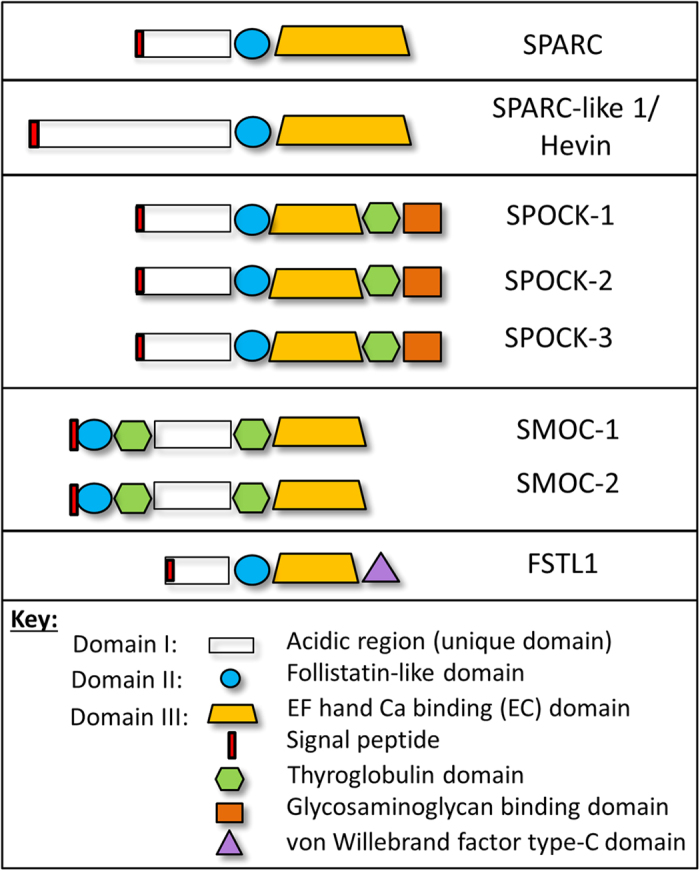
Domain structure of the SPARC family of proteins. The SPARC family of proteins share three main domains: domain I- a highly acidic region with low affinity calcium binding, domain II- a follistatin-like domain containing kazal-like serine protease inhibitor domains, and domain III- a calcium binding EF hand domain (also referred to as the EC domain). The signal peptide (indicated by red boxes) is typically located in domain I except for SMOC proteins. Individual members of the SPARC family also have distinct domains: SPOCK proteins contain a thyroglobulin domain and a glycosaminoglycan binding domain, while SMOC proteins contain 2 thyroglobulin domains. FSTL-1 contains a von Willebrand factor type-C domain.

**Figure 2:**
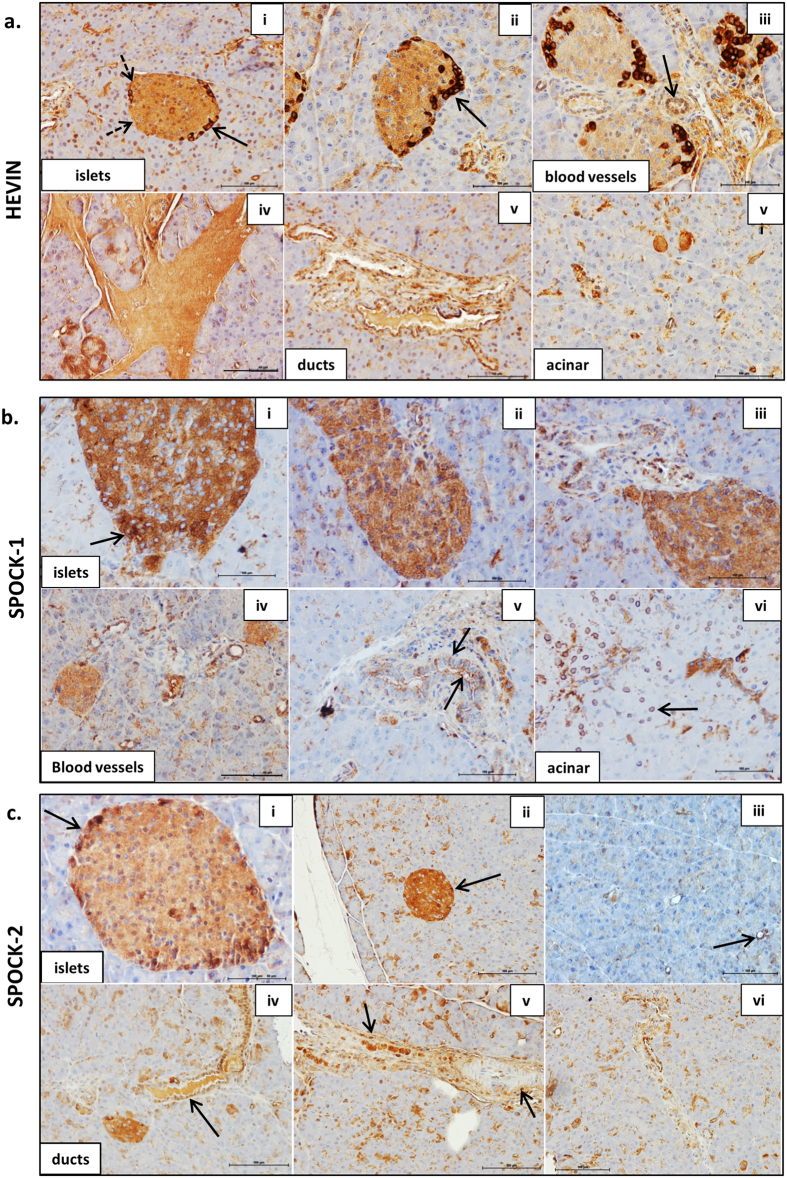
The SPARC family of proteins are widely expressed in the pancreas. ICR mouse pancreas sections were probed with antibodies to: Hevin (**a**), SPOCK-1 (**b**), and SPOCK-2 (**c**), followed by ABC-DAB staining (brown) and counterstained with haematoxylin (blue). Images are representative of 3–5 islets and ducts per section acquired using a 20X objective. Scale bar 100 μm. n = 2–3 independent experiments from 3 different mouse pancreas. See main text for explanation of arrows.

The SPARC family are defined as secreted matricellular proteins and contain signal peptide sequences to target them to the secretory pathway, as indicated in Fig. 1 (confirmed by Phobius database). They were therefore expected to be observed extracellularly. While Hevin, SPOCK-1 and FSTL-1 staining was observed at the cell surface, all members of the SPARC family also demonstrated cytoplasmic staining, and in some cases staining in the nucleus could also be observed. Cytoplasmic staining of SPARC family proteins has been previously described for Hevin^72^, SPOCK-1^73^ and SMOC-2^59^,^74^. It is likely that all SPARC family proteins are present in the extracellular environment, but this is not easily observed by immunohistochemistry when there is extensive cytoplasmic staining. However, the staining we observe clearly demonstrates the presence intracellularly of all SPARC family proteins. It will therefore be important to consider a possible intracellular role for these proteins in addition to their function in the extracellular matrix. One potential explanation for the intracellular location of these proteins is expression of splice variants lacking the signal peptide, and this is explored further below.

In summary, Hevin, the SPOCKs and the SMOCs were strongly expressed throughout islets consistent with expression in β cells. Furthermore, Hevin, SPOCK-2, and SMOC-2 showed stronger staining in cells at the islet periphery, consistent with higher expression in α cells. All SPARC family proteins were detected in ductal cells, while SPOCK-1, -2, -3, SMOC-1, and FSTL-1 were found also in ductal basement membranes. Hevin, the SPOCKs, the SMOCs and FSTL-1 were all found in selected acinar cells and in blood vessels throughout the pancreas. FSTL-1 on the other hand was not strongly expressed in islet parenchymal cells, but instead staining was consistent with expression primarily in islet basement membranes and blood vessels. This suggests that FSTL-1 is likely to be primarily expressed by stromal cells such as fibroblasts and endothelial cells, and the FSTL-1 staining pattern is highly reminiscent of SPARC expression^28^. The SPARC family of proteins are therefore clearly expressed in the pancreas, specifically in islet cells, stromal cells and pancreatic ducts, and warrant further investigation as to their function within the pancreas.

### Identification of multiple isoforms of the SPARC family and their expression in specific pancreatic cell types

In order to confirm the expression of SPARC family proteins observed in pancreas sections, we analysed expression in specific cell types by western blot. Protein quantification was performed to ensure equal loading. Given that cell lysates were obtained from different cell types with variable actin expression it was not possible to standardise the samples to compensate for small variations in loading, and therefore only a qualitative analysis was performed.

Hevin was expressed in both INS-1 and MIN-6 β cells (Fig. 5a), in agreement with the islet staining pattern observed using immunohistochemistry, and was also expressed by stromal cells such as PS-1 stellate cells, MRC5 fibroblasts and HUVEC endothelial cells, consistent with staining in basement membranes and blood vessels. SPOCK-1, -2, and -3 were also clearly detectable in both β cell lines examined (Fig. 5b–d), consistent with staining in islet cells shown by immunohistochemistry, and were also expressed in stromal cells such as stellate cells, fibroblasts and endothelial cells. Although only weakly detected in MIN-6 cells, SMOC-1 was strongly expressed in INS-1 β cells (Fig. 5e), consistent with the IHC staining. SMOC-1 was also detected in endothelial cells, but is either absent or very weakly expressed in fibroblast and stellate cells. SMOC-2 on the other hand was not detected by western blotting (data not shown). FSTL-1 was not detected in β cells, consistent with the absence of staining in the majority of islet cells by immunohistochemistry. Instead, FSTL-1 was primarily detected in PS-1 stellate cells, with weak expression in fibroblasts and endothelial cells (Fig. 5f) consistent with basement membrane and blood vessel-like staining in pancreas sections.

A further striking observation in the series of western blotting experiments in Fig. 5 is the presence of multiple bands for all SPARC family proteins, with the exception of FSTL-1. We also observed clear evidence of cell-type specific expression of these isoforms. For example, amongst the cell types examined endothelial cells uniquely express a 65 kDa variant of SMOC-1. Similarly, only pancreas-derived cells expressed a 110 kDa isoform of Hevin, while β cells uniquely express high levels of an additional 39 kDa isoform. A summary of molecular weights detected for each protein is shown in Suppl. Table 1. As far as we are aware this is the first systematic analysis of multiple isoforms of the extended SPARC family of proteins. The identity of these isoforms and the mechanisms underlying the cell-type specific expression are not known. However, cell type specific expression of isoforms may well in part explain some of the contradictory and controversial effects of the SPARC family of proteins on cell function, and their often complex association with clinical diseases^75^.

Possible explanations for the observation of multiple isoforms include: (1) post-translational modifications such as phosphorylation, glycosylation and addition of glycosaminoglycans, (2) protein cleavage into peptide fragments^46^,^47^, (3) expression of alternative splice variants, (4) the use of alternative translational start sites^76^ and (5) cross-linking by transglutaminase^77^,^78^. We therefore performed a systematic analysis of these factors for the wider SPARC family of proteins, combining both bioinformatics and experimental approaches.

### Post-translational modification of the wider SPARC family of proteins

Potential glycosylation and phosphorylation sites for each extended family of SPARC protein were identified using GeneCards, UniProt, and Phosphosite Plus. As shown in Suppl. Table 2, Hevin can be extensively modified, with 12 potential glycosylation sites and 8 phosphorylation sites. The SPOCKs also contain sites for both glycosylation and phosphorylation, and are known to contain O-linked glycosaminoglycans at serine residues in the C’ terminal region (Suppl. Table 2 and Figure 1). Glycosaminoglycan linkage can increase the molecular weight by 20 kDa or more^79^. SMOC-1 can be extensively modified through 9 glycosylation sites, compared to SMOC-2 with only 2 sites. Lastly, FSTL-1 has up to 3 sites for glycosylation and up to 5 for phosphorylation. All proteins in the extended SPARC family can therefore undergo varying degrees of post-translational modification.

### Alternative splicing of the SPARC family of proteins

We previously performed an initial analysis of alternative splice variation in matricellular proteins^80^. To determine whether the protein isoforms observed in Fig. 5 could be due to alternative splicing we further analysed the SPARC family splice variants banked in the ENSEMBL database. As shown in Suppl. Table 3, there is evidence of a large number of splice variants for the SPARC family of proteins. In particular, 17 coding variants were identified for SPOCK-3. However, since some coding sequences (CDS) were found to be incomplete, we restricted our further analysis to coding variants for which the complete CDS is known. Protein FASTA sequences were obtained for complete CDS transcripts and domain structures were predicted using the InterPro database. As shown in Fig. 6, in many cases multiple splice variants encode highly similar proteins. However, for each of the extended SPARC family of proteins at least one alternative splice variant with distinct protein sequence was identified. In particular, for Hevin, SPOCK-1, SPOCK-3 and SMOC-2 at least one alternative variant lacking the signal peptide was identified, suggesting that both intracellular and extracellular isoforms of these proteins exist. The intracellular isoforms may explain the cytoplasmic staining for these proteins described in Fig. 1. It is also possible that intracellular isoforms exist for SPOCK-2, SMOC-1 and FSTL-1 for which the complete sequence is not yet known and was therefore not included in this analysis.

Perhaps the most striking difference between isoforms of the extended SPARC family is the size of the acidic domain I, suggesting functional significance of domain I variation. However, the role of this domain is not well understood. Domain I is known to bind to calcium but with low affinity compared to the EC domain, and in SPARC domain I is involved in the regulation of cell migration^81^. In SPOCK-3, the acidic domain I is involved in MT-MMP inhibition^48^ while in SPOCK-2 it is involved in its regulation of SPOCK-3^49^. This domain has diverged and acquired additional acidic residues during evolution^82^,^83^,^84^,^85^. It is also the least conserved domain between different SPARC family proteins, and is the primary feature that distinguishes SPARC from Hevin. Domain I may therefore confer diversity of function to each SPARC family protein, and this diversity is then further expanded by alternative splicing. This analysis therefore suggest the functional importance of domain I variation in the SPARC family of proteins.

### Hevin isoforms – analysis of post-translational modification and splice variants

As described above, cell-specific expression of 110 kDa and 39 kDa Hevin isoforms was observed in addition to a widely expressed 49 kDa isoform. The predicted molecular weight of the Hevin precursor protein encoded by the primary transcript is 75 kDa, and the known Hevin splice variants for which the complete CDS is known are unlikely to explain the isoforms observed (Suppl. Table 3). However, 39–49 kDa Hevin bands have been reported to arise from cleavage by ADAMTS4 and MMP3, and Hevin is also a substrate for thrombin and plasmin digestion^46^,^47^. These low molecular weight Hevin proteins (39 and 49 kDa) are therefore likely to represent the products of enzymatic cleavage. The presence of the 39 kDa fragment exclusively in β cells suggests additional proteolytic cleavage of Hevin in these cells, perhaps reflecting a β cell specific role for Hevin fragments.

High molecular weight Hevin-reactive bands have also been previously observed^44^,^45^,^46^,^86^. Based on the number of predicted glycosylation sites (Suppl. Table 2), we hypothesized that the 110 kDa band is likely to reflect extensive glycosylation. To test this, we digested PS-1 stellate cell lysates with PNGase-F to test for the presence of N-linked glycosylation. As shown in Fig. 7a, an additional band at 36 kDa appeared following de-glycosylation. However, only the 49 kDa band and not the 110 kDa band showed any detectable decrease in intensity. The Hevin antibody used in these experiments recognises an epitope in the N-terminus and would therefore be predicted to recognise N-terminal cleavage products. These results therefore suggest that the 49 kDa band is a 36 kDa N-terminal cleavage product with approximately 15 kDa of N-linked glycosylation. The full length 110 kDa isoform may be conformationally resistant to glycosylase treatment or consist primarily of O-linked glycosylation. Alternatively, Hevin is predicted to be a substrate of transglutaminase (TRANSDAB)^87^, and the 110 kDa band may therefore represent oligomer formation due to cell-type specific cross-linking. Supporting this hypothesis, SPARC is also known to form oligomers as a result of transglutaminase-mediated cross-linking^77^,^88^.

Although no direct evidence of Hevin splice variants was observed in these experiments, subsequent experiments using an alternative Hevin antibody recognising a C’ epitope revealed the presence of an additional third isoform in PS-1 cells at approximately 60 kDa (Suppl. Figure 1), whereas only two isoforms were detected in PS-1 cells with the N’ antibody. This observation could suggest the presence of an alternative splice variant lacking the N-terminus. Consistent with this, Hevin variant 005 has a predicted molecular weight of 62 kDa (Suppl. Table 3). Since this variant lacks a portion of the N-terminal region it may exist in a conformation that is not recognised by the N-terminal antibody used. Variant 005 lacks a signal peptide and is predicted to be an intracellular isoform. However, mRNA studies would be required to confirm the detection of this potentially novel intracellular Hevin splice variant.

Proteolytic cleavage of Hevin has been shown to produce a “SPARC-like fragment”^46^,^47^ that is likely to correspond to, or be contained within, the approximately 50 kDa band detected using the C-terminal Hevin antibody (Suppl. Figure 1). It has been suggested that this SPARC-like fragment may compensate for the loss of SPARC expression^44^,^46^,^89^. SPARC can be suppressed for example as a result of SPARC promoter methylation during tumourigenesis^90^. Furthermore, SPARC and Hevin have overlapping and compensatory roles in angiogenesis inhibition^91^. We therefore tested whether reducing SPARC expression in PS-1 stellate cells by siRNA knockdown results in a compensatory increase in the presence of proteolytic cleavage products detected by both the N’- and C’-antibodies. As shown in Fig. 7b–e, despite achieving 90% knockdown of SPARC expression, no significant change in the detection of either the full length Hevin or smaller fragments was observed with either the N-terminal or C-terminal antibodies.

Therefore, although previous reports have suggested that C-terminal Hevin fragments may compensate for loss of SPARC expression, we did not find evidence to support this hypothesis within the cell types examined. However, the various isoforms of Hevin that we have observed are likely to have distinct properties and to fulfill specific functions within the particular cell types where they are expressed, and there is evidence for the importance of additional proteolytic cleavage products specifically in β cells.

### SPOCK protein isoforms

For each of the SPOCK proteins three different isoforms were observed. In the case of SPOCK-1, while an isoform of the predicted molecular weight (49 kDa) was observed in all cell lines examined, two additional isoforms (56 kDa and 100 kDa) were observed specifically in pancreatic stellate cells and β cells (Fig. 5b). SPOCK-1 has previously been detected in the 130–150 kDa range in human plasma and in kidney cells, and the increase in molecular weight is most likely due to the addition of large glycosaminoglycan chains (both chondroitin and heparin sulphate chains) at residues 383 and 388 of the C-terminus (Suppl. Table 2)^79^,^92^. The detection of SPOCK-1 at 49 kDa in all cell types suggests a native unglycosylated form of SPOCK-1 is also widely produced, and the addition of glycosaminoglycans in specific cell types may create novel functions. A 33 kDa intracellular alternative splice variant was also identified in ENSEMBL (Suppl. Table 3 and Figure 6), and it is also possible that the observed 49 kDa and 56 kDa isoforms represent glycosylated forms of this variant.

Multiple splice variants of SPOCK-2 were also identified in ENSEMBL, primarily encoding variants highly similar to the primary transcript, with the exception of variant 202 that encodes a small (8 kDa) protein containing a signal peptide but lacking any conserved domains (Fig. 6). However, proteins of such low molecular weight would not be observed by our western blot analysis. Proteins of around the expected molecular weight of the primary and similar transcripts (47 kDa) were observed in fibroblast and endothelial cells, though not in pancreatic stellate and β cells (Suppl. Table 1 and Figure 5c). In contrast, in β cells SPOCK-2 was detected at 60 kDa and 120 kDa. As shown in Suppl. Table 2, SPOCK-2 contains both glycan and glycosaminoglycan binding sites, and variable glycosylation is therefore likely to explain the larger isoforms observed specifically in β cells. Interestingly, pancreatic stellate cells express very low levels of SPOCK-2.

**Figure 3:**
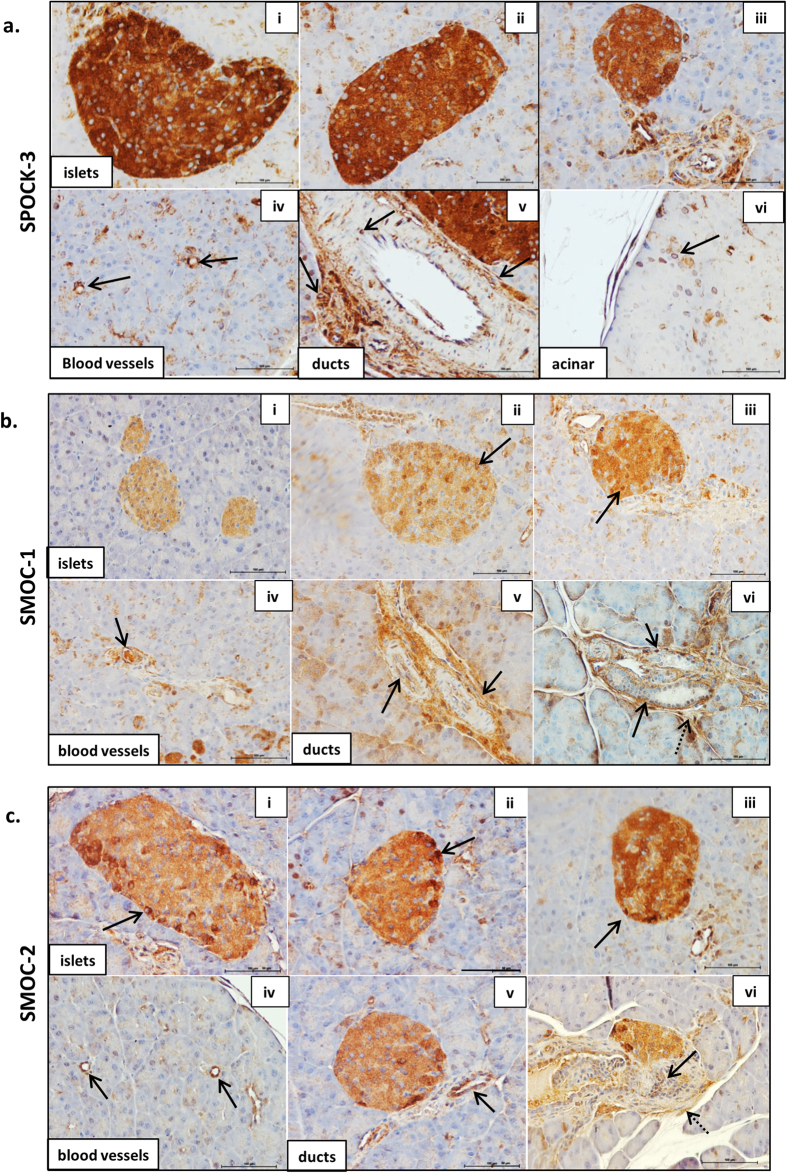
The SPARC family of proteins are widely expressed in the pancreas. ICR mouse pancreas sections were probed with antibodies to: SPOCK-3 (**a**), SMOC-1 (**b**), SMOC-2 (**c**). See Figure legend 2 for additional details.

For SPOCK-3 a large number of alternative transcripts were identified in ENSEMBL, with a total of 13 protein-coding variants for which the complete coding sequence is known (Suppl. Table 3). Of these, multiple transcripts are predicted to produce a 49 kDa protein that is highly similar to the primary transcript in terms of predicted domain structure (001, 002, 006, 012, 014, 015, 016), as shown in Fig. 6. Interestingly, variants lacking certain domains or with additional domains were also identified, encoding proteins of varying molecular weights between 36 and 44 kDa. For example, transcript 005 lacks the thyroglobulin domain and C’ glycosaminoglycan binding domain, while transcript 010 lacks the follistatin domain (Fig. 6). An alternative splice variant of SPOCK-3 missing the thyroglobulin domain and glycosaminoglycan binding sites has been previously described in kidney cells and glioma, referred to as N-Tes, that is likely to correspond to variant 005 in ENSEMBL^48^. SPOCK-3 transcript 013 contains a second thyroglobulin domain situated before the EC domain, almost *pseudo*-SMOC-like in structure. Interestingly, the thyroglobulin domain is involved in the IGF binding properties of IGFBPs, as well as protease inhibitory functions^93^,^94^,^95^,^96^, and it will therefore be interesting to test the function of splice variants either lacking (005) or with additional (013) thyroglobulin domains. SPOCK-3 splice variants also contain extensive variation within the acidic N-terminus, and this domain varies from just 12 amino acids in transcript 202, compared to 129 amino acids in the primary transcript. Importantly, transcripts 201, 017 and 202 all lack the signal peptide and therefore are predicted to be intracellular proteins, consistent with the intracellular staining we observed in Fig. 3.

Since SPOCK-3 can also be glycosylated (Suppl. Table 2), the 65 kDa stromal isoform is likely to correspond to a glycosylated form of the 49 kDa protein encoded by the primary or similar transcripts. In order to test whether the variants we observe are due to glycosylation, we subjected PS-1 cell lysates to PNGase-F digestion. As shown in Fig. 8a, the 34 kDa isoform increases in intensity by at least four fold following digestion, suggesting that this isoform is present in both N-linked glycosylated and unglycosylated forms, and that the 34 kDa isoform is the unglycosylated form. Furthermore, a second band of 45 kDa also appears following digestion, suggesting the presence of a second distinct protein isoform that is normally N-glycosylated. The size of this band is consistent with the primary transcript, or an alternative transcript of similar size. These experiments demonstrate the presence of at least two isoforms of SPOCK-3 in stromal cells, most likely representing the primary 49 kDa transcript that exists in a glycosylated form and variant 005/N-Tes that can exist in both glycosylated and unglycosylated form (34–37 kDa). In contrast, only a single isoform is observed in β cells. The glycosylation detected by PNGase F treatment may well represent N-linked glycosaminglycan chains attached to non-consensus N-glycosylation motifs. N-linked glycosylation at non-consensus motifs are now known to occur in mammalian genomes^97^. Multiple high molecular weight bands (>90 kDa) were observed in PS-1 and β cell lysates at variable intensities, that are likely to reflect the addition of glycosaminoglycan chains (Suppl. Figure 2d). Mouse SPOCK-3 was recently shown to contain heparin sulphate proteoglycans, although N-linked glycosylation was not detected in mouse SPOCK-3^98^. These experiments therefore demonstrate the novel finding that SPOCK-3 contains novel N-linked glycosylation sites.

As described above, the observation that two bands appear following de-glycosylation suggests the expression of two distinct core proteins, and is consistent with the presence of 005/N-Tes (~36 kDa) plus the full-length protein corresponding to the primary transcript or similar (~49 kDa) in PS-1 cells. However, other explanations are possible, including the presence of digestion products or differential post-translational modification in addition to N-linked glycosylation. We therefore sought to test whether distinct splice variant transcripts could be detected.

The exon structure of all 17 SPOCK-3 alternative splice variants banked in ENSEMBL was examined in order to design primers to detect variants. SPOCK-3 is unusual in that alternative splicing affects multiple regions across the entire coding sequence, as well as the 5′ UTR, suggesting that SPOCK-3 has a high degree of tolerance for variability within the protein structure^80^. Variants truncated at the 5′ end (201, 017, 202), at the 3′ end (005), and in internal regions (013, 010, 202, 018) are described in the database. As shown in Suppl. Figure 3, 19 exons are currently identified, the first 4 of which form the 5′ UTR. The 7 transcripts that encode proteins highly similar to the primary transcript are shown in blue. It can be seen that these variants differ primarily in the 5′ UTR exons, with 001/014, 002/015 and 006/16 pairs having identical coding sequences, differing only in the use of 5′ UTR exon 1/exon 2. In contrast, 012 uses 5′ UTR exon 4 which in fact contains an upstream translation start producing a predicted protein with an additional 12 amino acids at the N-terminus (translation performed using exPASY). However, it is not clear whether this start codon is used. Interestingly, exons 8 and 9 (as labelled in Suppl. Figure 3) are identical microexons of 9 bp. The coding sequences of 001, 014, 002 and 015 are therefore identical, and differ from 006/016 by only 3 amino acids. The significance of microexons is not fully understood, but they have been shown to have functional effects^99^,^100^, and have been previously identified in murine SPOCKs^98^. Since these 7 transcripts produce highly similar proteins, we focused on transcripts predicted to encode medium length (201, 013) and short (017, 010, 202, 005) proteins for experimental confirmation.

Specific RT-PCR primers were designed to each of these variants, with the exception of transcript 017 for which unique primers could not be designed. The primer locations are shown in Suppl. Figure 3 and the sequences and predicted product sizes given in Suppl. Table 4. Generic primers were designed that should size differentiate between transcript 201 and the remaining transcripts (GenA), and that should amplify all transcripts listed except for 005 and 010 (GenB). As shown in Fig. 8b, we detected the 005 transcript in PS-1 cells, and the specificity of the PCR product was confirmed by Sangar sequencing. Transcripts 010, 201 and 202 were not detected using the primers specific to these sequences. The generic primers GenA and GenB detected PCR products of the expected size, and specificity was confirmed by Sangar sequencing. Since the GenB primers could not amplify 005 this confirms the presence of at least one other transcript in addition to 005, most likely the primary variant. The sequence of the GenA PCR product revealed the absence of microexons 8 and 9, and the sequence was instead identical to variants 006/016 (which have identical CDS). We therefore demonstrate that pancreatic stellate cells express two distinct SPOCK-3 splice variants, 005/N-Tes and 006/016, corresponding to the deglycosylated ~34 and ~45 kDa proteins observed by western blot.

### Isoforms of the SMOC proteins

Two distinct isoforms of SMOC-1 were identified, with highly cell type specific expression (Fig. 5 and Suppl. Table 1). The 65 kDa isoform was expressed uniquely in endothelial cells, while the 53 kDa isoform was present primarily in β cells. Interestingly, SMOC-1 was either not detected or only weakly detected in fibroblasts and stellate cells. Only two splice variants with complete CDS were identified by bioinformatics analysis and both encode for highly similar 48 kDa proteins (Suppl. Table 3 and Figure 6), suggesting that alternative splicing is unlikely to account for the two isoforms observed. However, SMOC-1 has 9 predicted glycosylation sites (Suppl. Table 2) and therefore alternative glycosylation is more likely to explain the two distinct isoforms identified, as suggested by PNGase digestion in studies elsewhere^54^,^101^,^102^. It will therefore be important to analyse the effect of alternative glycosylation on SMOC-1 protein function to understand the roles of distinct isoforms in endothelial cells and β cells.

For SMOC-2, three alternative transcripts were identified in ENSEMBL: transcripts 001 and 002 are predicted to encode proteins of similar molecular weight (~50 kDa) and with overall similar domain structure, with the exception of a truncated acidic domain in 001 (Suppl. Table 3 and Figure 6). In contrast, variant 201 is predicted to encode a small 14 kDa protein that lacks a signal peptide and any predicted functional domains. The coding region of variant 201 is in fact not overlapping with that of the primary transcript and is therefore likely to encode an intracellular protein of quite distinct function. SMOC-2 has been previously detected between 54 kDa and 60 kDa, and was shown to be glycosylated by PNGase digestion^74^,^101^.

SMOC proteins have been associated to many cancers and reported to be involved in cellular differentiation, cell-cycle progression and in regulating cell responses to the environment. However, SMOC proteins are largely unstudied in pancreatic diseases such as PDAC and diabetes. We have shown that SMOC-1 and SMOC-2 are widely expressed in the pancreas, and that specific SMOC-1 isoforms are expressed in endothelial cells and β cells. It will therefore be important to study the function of SMOC isoforms in these cells and in pancreatic disease.

### Isoforms and expression of FSTL-1

**Figure 4:**
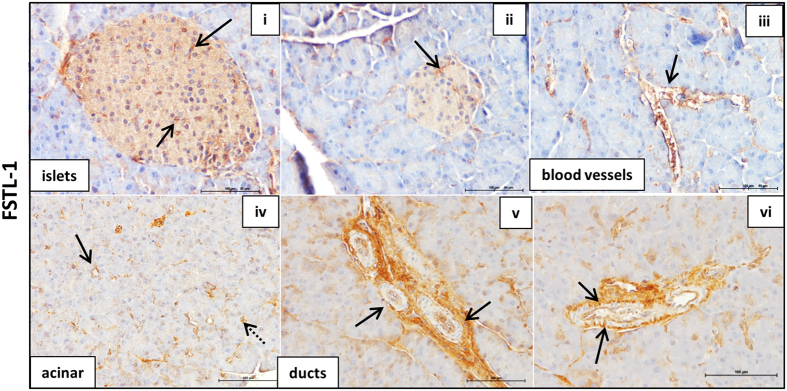
The SPARC family of proteins are widely expressed in the pancreas. ICR mouse pancreas sections were probed with antibodies to FSTL-1. See Figure legend 2 for additional details.

**Figure 5:**
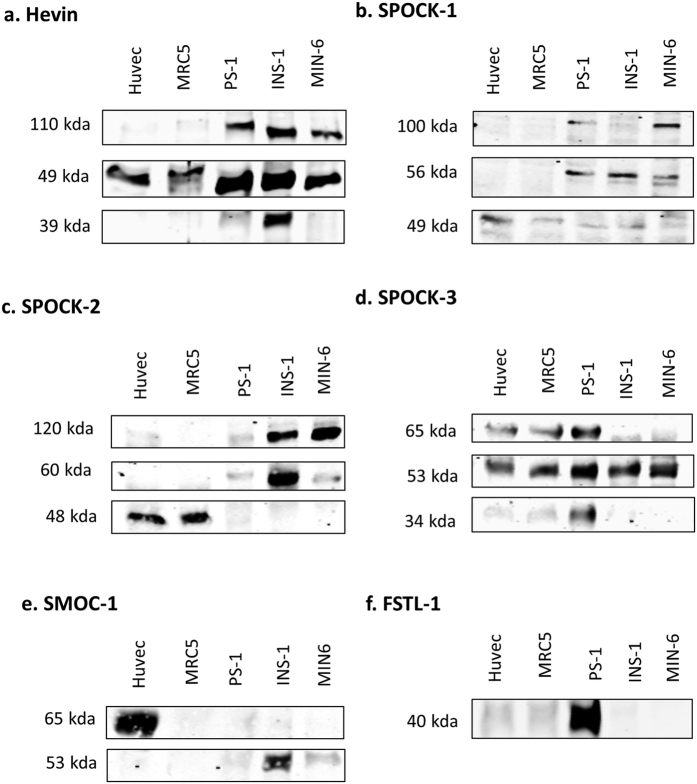
Identification of multiple isoforms of the SPARC family and their expression in specific cell types. For each of the indicated cell types 20–25 μg of protein lysate was analysed by western blot using antibodies to Hevin (**a**), SPOCK-1 (**b**), SPOCK-2 (**c**), SPOCK-3 (**d**), SMOC-1 (**e**), FSTL-1 (**f**). Blots were cropped to show consistent bands observed in at least 2 independent experiments. Full, uncropped blots are shown in Suppl. Figure 2.

**Figure 6:**
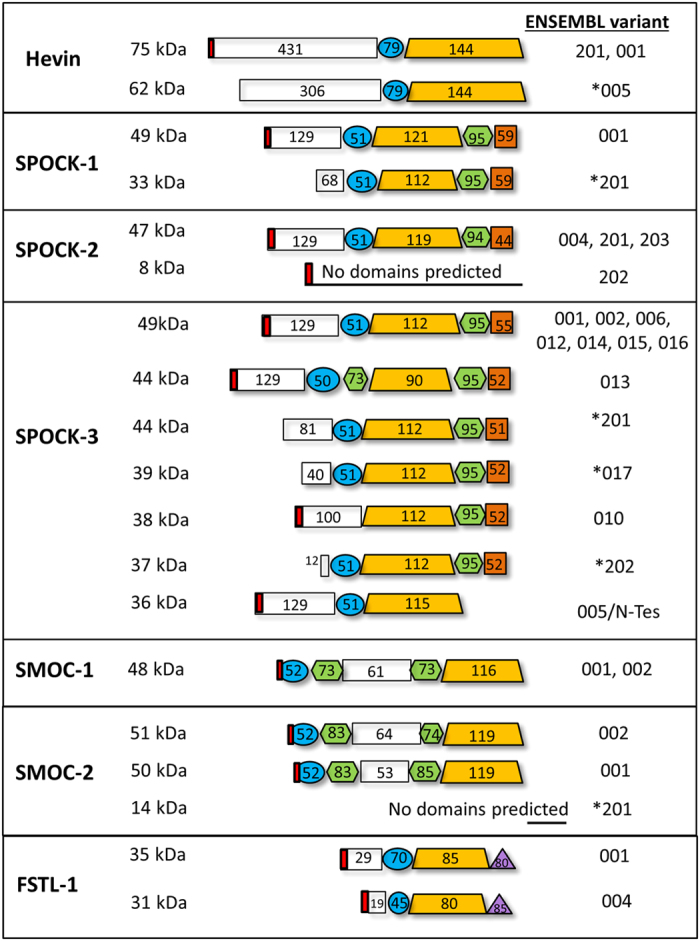
Predicted domain structures of alternative splice variants of the extended SPARC family. FASTA format protein sequences of the SPARC family alternative transcripts (complete CDS) were obtained from ENSEMBL and the InterPro database was used to determine domain structures. Numbers within domain structures indicate amino acid residues for each domain. Red boxes indicate the presence of a signal peptide. Alternative transcripts that do not contain the signal peptide are indicated with asterisks.

**Figure 7:**
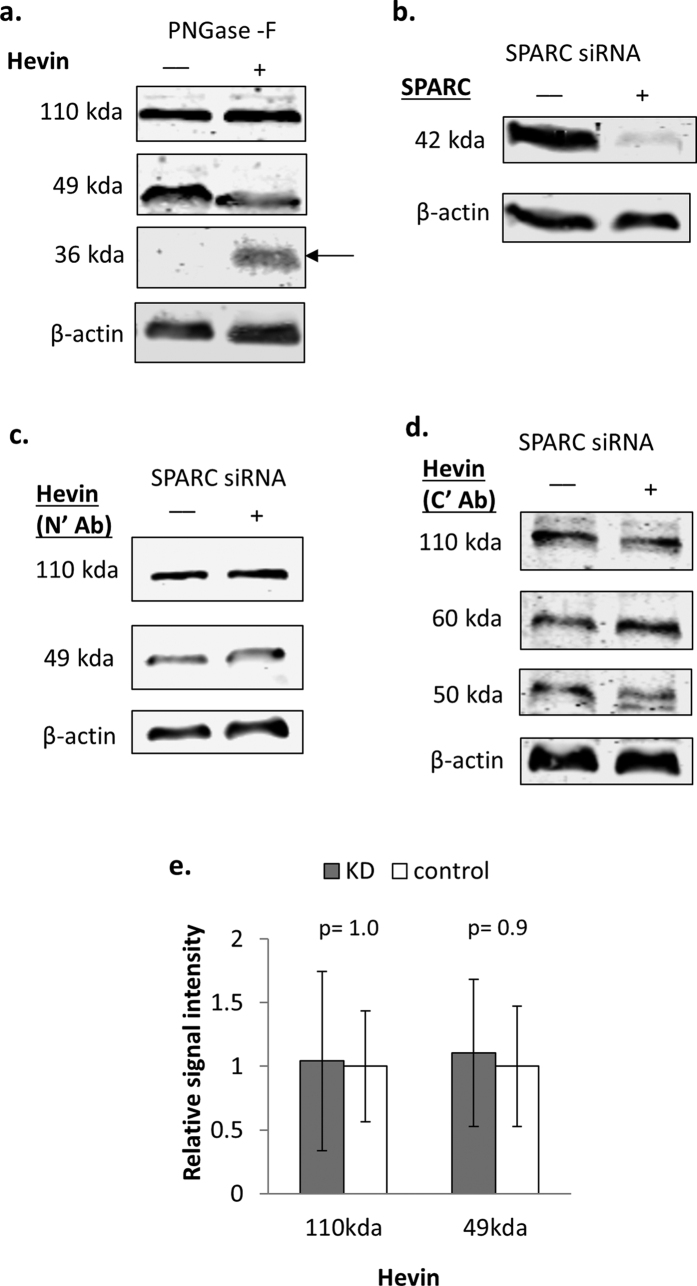
Hevin contains N-linked glycosylated isoforms, but expression is not regulated by loss of SPARC expression. (**a**) PS-1 cell lysates were treated with PNGase-F glycosidase and analysed by western blot using antibodies to Hevin and β actin as a loading control. Images are representative of 3 independent replicates, and the mean molecular weight observed for each band is indicated next to the blots. Arrow indicates de-glycosylated band. (**b–e**) PS-1 stellate cells were treated with anti-SPARC (+) or control (−) siRNA for 48 hours. Cell lysates were then analysed by western blot using antibodies to SPARC to confirm knockdown (**b**), Hevin N-terminus (**c**), or Hevin C-terminus (**d**). On average 90% knockdown of SPARC expression was achieved in the three experiments performed (+/− 6%). In (**e**), the graph shows mean signal intensity +/− SEM for the Hevin N-terminal antibody, standardised to β actin and relative to the control, for cells treated with SPARC (KD) or control siRNA. Images are representative of 3 independent replicates, and statistical significance was measured using the Student’s t-test (unpaired, two-tailed). p-values are indicated in the graph. Uncropped blots are shown in Suppl. Figure 4.

**Figure 8:**
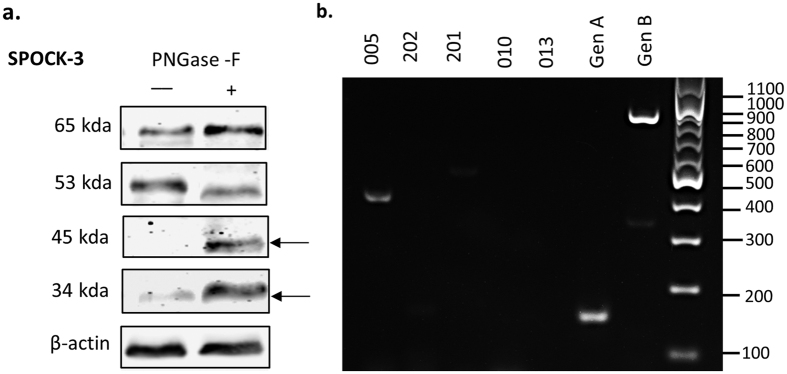
Expression of SPOCK-3 splice variants in pancreatic stellate cells. (**a**) PS-1 cell lysates were treated with PNGase-F glycosidase and analysed by western blot using antibodies to SPOCK-3 and β actin as a loading control. Images are representative of 3 independent replicates, and the mean molecular weight observed for each band is indicated next to the blots. Arrows indicate de-glycosylated bands. Uncropped blots are shown in Suppl. Figure 5. (**b**) PS-1 mRNA was isolated and expression of the indicated SPOCK-3 splice variant expression was analysed by RT-PCR.

Expression of FSTL-1 was the most highly specific of the SPARC family proteins. We detected strong expression of a single ~40 kDa isoform specifically in pancreatic stellate cells, with weaker expression in fibroblasts and endothelial cells (Fig. 5 and Suppl. Table 1). This is consistent with the stromal expression pattern and staining in basement membranes observed by immunohistochemistry (Fig. 4). We identified two alternative transcripts with complete CDS in ENSEMBL that are predicted to share the same functional domains except for a truncated acidic domain in variant 004 (Suppl. Table 3 and Figure 6). Both variants contain a signal peptide sequence and are therefore predicted to be secreted extracellular proteins. The molecular weight of the observed protein (40 kDa) is consistent with the predicted molecular weight of the primary transcript with minor post-translational modification such as glycosylation, as has been previously reported^103^. FSTL-1 is sometimes overlooked as a member of the SPARC family since it has the least structural and sequence homology to SPARC and it has been suggested that the calcium binding EF hand in FSTL-1 may be non-functional^103^. Our data shows that FSTL-1 is expressed by stromal cells within the pancreas with an expression pattern highly reminiscent of SPARC^28^, and is expressed at high levels specifically by pancreatic stellate cells. This suggests that FSTL-1 may play a related role to SPARC in pancreatic disease and that the function of FSTL-1 warrants further study.

### Despite SPARC-like expression pattern, exogenous FSTL-1 does not regulate β cell growth or proliferation

**Figure 9:**
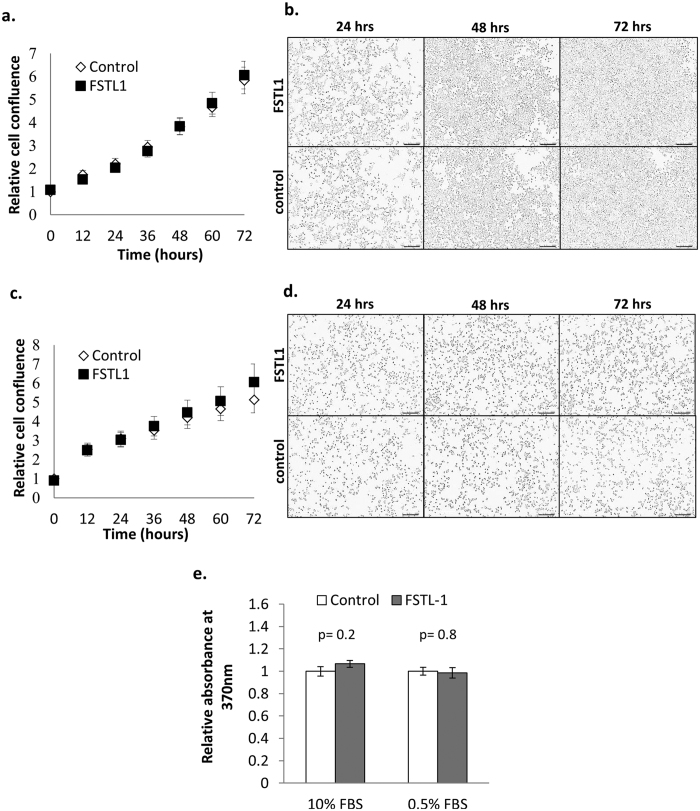
FSTL-1 does not regulate β cell growth and proliferation. INS-1 β-cells were plated at a density of 1.5 × 10^4^ cells/well in a 96 well plate. Post synchronisation, the cells were either provided with untreated medium or medium supplemented with 100 ng/ml rFSTL-1 in either 10% FBS (**a**,**b**) or 0.5% FBS (**c**,**d**) and cultured for a further 72 hrs. Cell growth was monitored every 12 hrs during this period using the IncuCyteZOOM live cell imaging system. In (**a**,**c**) graphs shows mean cell confluence relative to control +/− SEM, n = 30 from 5 independent experiments, while representative images taken at the indicated timepoints are shown in (**b**,**d**). BrdU incorporation was measured for the last 24 hrs of the 72 hr culture, and the graph in (**e**) shows mean absorbance relative to the control +/− SEM. Statistical significance was measured using Student’s t-test (unpaired, two-tailed) and p-values are indicated in the graph.

Compared to other SPARC family proteins that we have shown to be highly expressed throughout islets and in β cells, FSTL-1 and SPARC are the only SPARC family proteins detected in stromal cells and not in β cells by western blotting and IHC (Figs 4 and 5 and ref. 28). Like SPARC, FSTL-1 has been shown to regulate signalling of the TGF-β superfamily and to regulate growth factor signalling^28^,^39^,^60^,^61^,^104^,^105^. We therefore tested whether FSTL-1 can similarly regulate β cell growth. INS-1 β cells were treated with rFSTL-1 over a period of 3 days. Cell growth was measured using the Incucyte Zoom live cell imaging system, and cell proliferation was measured by BrdU incorporation over the final 24 hour period. However, as shown in Fig. 9, the addition of exogenous FSTL-1 had no effect on the growth or proliferation rate of β cells. Despite a highly similar pattern of expression to SPARC, FSTL-1 is therefore unlikely to be directly involved in the regulation of islet growth and survival.

### FSTL-1 inhibits cancer cell proliferation

**Figure 10:**
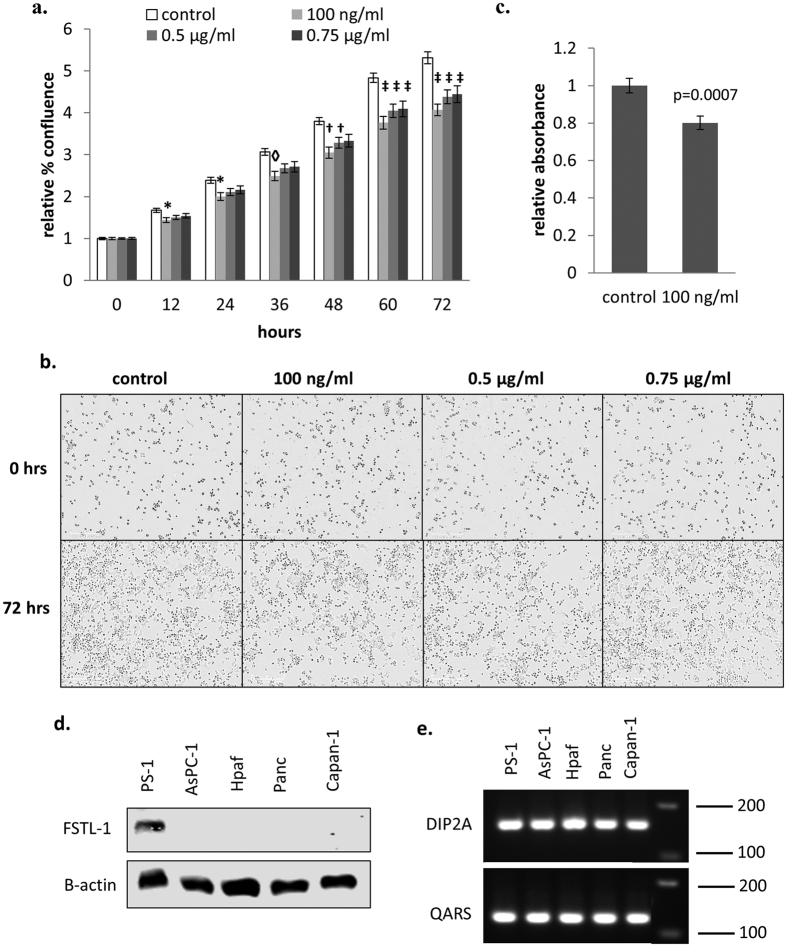
FSTL-1 inhibits pancreatic cancer cell growth and proliferation. AsPC-1 cancer cells were plated at a density of 5 × 10^3^ cells/well in a 96 well plate. Post synchronisation, the cells were cultured in complete media (10% FBS) containing the indicated concentrations of rFSTL-1 for 72 hrs. Cell growth was monitored every 12 hrs during this period using the IncuCyteZoom live cell imaging system. (**a**) Graph shows mean cell confluence relative to the control +/− SEM, n = 17–18 data pooled from 3 independent experiments, and representative images taken at 72 hrs are shown in (**b**). BrdU incorporation was measured for the last 24 hrs and the graph in (**c**) shows the mean absorbance relative to the control +/− SEM n = 16–17 data pooled from 3 independent experiments. Statistical significance was measured using one-way ANOVA and p-values are indicated in the graph (*p < 0.05, ^◊^p < 0.01, ^†^p < 0.001, ^‡^p < 0.0001). (**d**) FSTL-1 expression in pancreatic cancer cells was analysed by western blotting. Uncropped blots are shown in Suppl. Figure 6. (**e**) RT-PCR was performed on the indicated cell lines using primers specific for DIP2A, or QARS as a housekeeping gene. A single PCR product of the expected size was observed for both primer pairs, and all negative controls were blank.

Although we did not observe any change in the growth or proliferation of β cells in response to FSTL-1, previous reports have suggested that FSTL-1 can act as a tumour suppressor in breast and ovarian cancer^63^. We therefore examined the effect of FSTL-1 on pancreatic cancer cell growth and proliferation. The addition of rFSTL-1 was found to inhibit the growth (Fig. 10a & b) and proliferation (Fig. 10c) of pancreatic cancer cells. Western blotting revealed that FSTL-1 is only expressed by pancreatic stromal cells and not by cancer cells (Fig. 10d). However, both stellate cells and cancer cells express DIP2A (Fig. 10e), which acts as a receptor for FSTL-1^106^,^107^. Analysis of FSTL-1 immunohistochemistry data in the Human Cancer Atlas database shows that FSTL-1 is expressed at ‘medium’ levels in the normal pancreas but is not detected in the majority of pancreatic cancer tissues analysed^108^. Furthermore, FSTL-1 is similarly reduced compared to normal tissue in a range of other cancers including liver, breast, renal and stomach cancer^108^. Together, this data suggests that FSTL-1 produced by stromal cells normally acts to inhibit pancreatic cancer cell growth, and that FSTL-1 expression is downregulated within pancreatic tumours. Whether this difference is clinically significant would require further analysis that is outside the scope of this study. However, the data suggests that FSTL-1 is a novel tumour suppressor in pancreatic cancer. Furthermore, SPARC and FSTL-1 produced by stromal cells have opposing effects on pancreatic cancer cell growth, and it will therefore be of interest in future studies to test whether the combined signature of SPARC overexpression and FSTL-1 inhibition is useful diagnostically.

## Conclusions

The SPARC family of matricellular proteins are important multifunctional regulators of cellular and matrix interactions. Our data shows that the extended SPARC family is expressed in pancreatic β cells, stromal cells and in ducts. We identified multiple isoforms with cell-type specific expression that arise from a complex mixture of post-translational modifications and expression of alternative splice variants. Furthermore, intracellular variants of the wider SPARC family were also identified, and these are likely to have distinct functions compared to secreted variants. We have further shown that FSTL-1 inhibits pancreatic cancer cell growth, suggesting that SPARC and FSTL-1 produced by stromal cells have antagonistic effects on cancer cell growth.

We have shown that taking a systematic and holistic approach to the study of the SPARC family of proteins and addressing the complexity of the different isoforms will be essential for understanding the function of these proteins and their role in health and disease, and is likely to represent an important challenge for the field. This study therefore provides a foundation for future research investigating the SPARC family in pancreatic diseases such as diabetes and cancer.

## Methods

### Animals

Pancreas sections for immunohistochemistry were obtained from adult male and female outbred ICR mice (21–25 g) from Harlan, Bicester, UK. Tissues were fixed in 10% NBF and embedded in paraffin. Animal maintenance was performed in accordance with the principles of laboratory animal care. No procedures covered by UK Home Office Regulations were carried out in this study.

### Immunohistochemistry

Paraffin embedded sections (5 μm) of ICR pancreas were deparaffinised in histoclear (National Diagnostics) or histochoice (Amresco) and hydrated with 100% and 70% ethanol. Blocking was performed using 10% normal horse serum for 30 minutes in a humidified chamber. Antibodies to the SPARC family of proteins were selected to ensure that the epitope was either within the unique acidic domain I, or outside of any highly conserved domains. Full details of the antibodies used and concentrations are given in Suppl. Table 5. Primary antibodies were incubated overnight, followed by incubation with relevant biotinylated secondary antibody for 1 hour at room temperature. Antibody binding was detected using ABC peroxidase kit with DAB substrate (Vector Labs). Sections were counterstained with haematoxylin. Adjacent sections were stained in parallel with the relevant secondary antibody only as a negative control, and this was blank in all experiments. Digital images were acquired using a Nikon Eclipse 80i microscope and analysed using ImageJ software.

### Cell Culture

PS-1 pancreatic stellate cells and MRC5 fibroblast cells were kindly provided by Professor Hemant Kocher from the Barts Cancer Institute, Queen Mary University of London. AsPC-1, Hpaf, Capan-1, Panc-1 pancreatic cancer cells and INS-1 β cells were kindly provided by Dr. Charlotte Edling from the Blizzard Institute, Barts and the London. MIN-6 β cells were kindly provided by Professor Peter Jones of King’s College London. HUVEC endothelial cells were kindly provided by Dr. Andrew Snabaitis at Kingston University, London.

PS-1, AsPC-1, Hpaf, Capan-1, and Panc-1 cells were grown in RPMI-1640 media supplemented with 2 mM L-glutamine, 100 μg/ml penicillin and streptomycin, and 10% FBS (Gibco). INS-1 β cells were grown in RPMI-1640 supplemented with 2 mM L-glutamine, 100 μg/ml penicillin and streptomycin, 1 mM sodium pyruvate, 10 mM HEPES buffer, 0.05 mM β-mercaptoethanol and 10% FBS. MIN-6 β cells and MRC5 fibroblasts were grown in DMEM media supplemented with 100 μg/ml penicillin and streptomycin and 10% FBS. HUVEC cells were grown in F12K media supplemented with 100 μg/ml penicillin and streptomycin, 0.1 mg/ml heparin, 50 μg/ml ECGS (Millipore), and 10% FBS. Cells were incubated at 37 °C in 5% CO_2_.

### Cell growth assays

INS-1 β-cells (1.5 × 10^4^ cells/well) and AsPC-1 (5 × 10^3^ cells/well) pancreatic cancer cells were plated in a 96 well plate and synchronised in low serum media (0.5% FBS) for 24 hrs. Post synchronisation, the cells were either provided with fresh untreated medium or with medium containing various concentrations of rFSTL-1 (R&D Systems) and cultured for further 72 hrs. Cell growth was monitored every 12 hrs during this period using the IncuCyteZOOM live cell imaging system (Essen Bioscience). BrdU incorporation (Roche Applied Science) was measured for the last 24 hrs of the 72 hr culture according to the manufacturer’s instructions. Statistical significance was calculated using Student’s t-test (unpaired, two-tailed) or one-way ANOVA and a p-value < 0.05 was considered significant.

### siRNA knockdown

Transfection complexes were formed using human SPARC siRNA (J-003710-10 Dharmacon) and HiPerFect (Qiagen) in serum-free medium for 20 minutes at room temperature. Human non-targeting siRNA was used as a control (Dharmacon). During this period PS-1 cells were plated at a density of 1 × 10^5^ cells per well in a 24 well plate. Transfection reagents were then added to the cells to give a final concentration of 80 nM siRNA. Transfection was carried out for 48 hours and knockdown was confirmed by western blot with SPARC antibody. Hevin isoforms were then detected using antibodies specific to the N- and the C-terminus (see Suppl. Table 5 for antibody details).

### Protein expression analysis by Western Blot

Cells were lysed with RIPA buffer (Sigma Aldrich) for 20 minutes on ice in the presence of protease inhibitors (Halt^TM^ Protease Inhibitor Single Use Cocktail). Lysates were subsequently cleared by centrifugation and the supernatant collected. Samples were run immediately after lysis to avoid potential issues with protein degradation^86^. For glycosylase experiments, cell lysates were denatured at 100 °C for 4 minutes then incubated with PNGase-F for one hour at 37 °C according to the manufacturer’s instructions (New England BioScience).

Protein concentration was determined using the BCA protein assay (Bio-Rad DC). Sample buffer was added to lysates after which equal protein was loaded onto 12% polyacrylamide gels and subjected to SDS-PAGE (20–25 μg of protein/well). Proteins were then transferred onto nitrocellulose membranes and blocked with 5% milk solution. Membranes were incubated overnight at 4 °C with the relevant rabbit SPARC family primary antibody (see Suppl. Table 5) and mouse β-actin antibody (1:2500, Abcam) then washed with Tween TBS. Anti-rabbit and anti-mouse secondary antibodies conjugated respectively to IR800 and IR700 infra-red dyes were used for detection. Membranes were visualised by infrared using a Li-Cor Odyssey CLx scanner. Molecular weight and signal intensity was measured using Li-Cor Image Studio. Image processing of greyscale images for figure preparation was limited to changes in brightness and contrast applied to the entire blot, and in some cases the image was flipped to invert the order, or the order of the wells changed for clarity and consistency. In all cases the images shown are an accurate representation of the original data. Cropped blots are used in the main paper and the full uncropped blots are shown in supplementary data. Statistical significance was calculated using Student’s t-test (unpaired, two-tailed) and a p-value < 0.05 was considered significant.

### mRNA expression analysis by RT-PCR

Total RNA was isolated from PS-1 cells using an RNeasy Mini Kit, including on-column DNase I treatment (Qiagen). Total RNA was quantified using a NanoVue™ Plus Spectrophotometer and RNA integrity confirmed using an Agilent Bioanalyzer 2100. RNA (700 ng) was reverse transcribed to cDNA using a RevertAid First Strand cDNA Synthesis Kit (ThermoFisher Scientific). PS-1 cDNA was amplified using DreamTaq Green PCR Master Mix (ThermoFisher Scientific). Primers were designed to SPOCK-3 splice variants banked in ENSEMBL (Accessed March 2013) using PrimerBLAST (See Suppl. Table 4 and Suppl. Figure 3). Primer sequences used for DIP2A were: Forward primer- GCAGATGGTGTCCCTGTGAAC Reverse primer- CTGATTTGGATCTGGTTGCTGA. At least one primer in each pair was designed to be exon spanning to avoid amplification of any residual genomic template. RT-PCR products were separated on a 2% agarose gel in TAE. QARS primers (for-TTCCGGTGTCTCTGCAATGG; rev- CTGCTGAGCCTGAGTAGCG) were used as a loading/positive control. All negative controls were blank (no RT cDNA, no template cDNA, and PCR dH20 control).

### Bioinformatics

The ENSEMBL database was used to identify alternative splice variants of the human SPARC family of proteins. Only protein-coding transcripts for which the complete coding sequence (CDS) is known were included in the analysis. For these transcripts, protein FASTA sequences were downloaded. The ENSEMBL transcript IDs used are given in Suppl. Table 3 [download date April 2015]. Respective product sizes for each complete transcript were calculated using the Protein Molecular Weight Bioinformatic tool from the Sequence Manipulation Suite. Domain structures for alternative transcripts were determined using the InterPro protein sequence analysis and classification database. Signal peptide expression was determined using Phobius signal peptide predictor. Genecards, UNIPROT, Phosphosite plus, and TRANSDAB were used to identify predicted post-translational modifications of the SPARC family (primary variants). The Human Protein Atlas was accessed July 2016.

## Additional Information

**How to cite this article**: Viloria, K. *et al*. A holistic approach to dissecting SPARC family protein complexity reveals FSTL-1 as an inhibitor of pancreatic cancer cell growth. *Sci. Rep.* **6**, 37839; doi: 10.1038/srep37839 (2016).

**Publisher's note:** Springer Nature remains neutral with regard to jurisdictional claims in published maps and institutional affiliations.

## Supplementary Material

Supplementary Data
