# Supplementary material for: A holistic approach to dissecting SPARC family protein complexity reveals FSTL-1 as an inhibitor of pancreatic cancer cell growth
Source: Sci Rep. 2016 Nov 25;6:37839. doi: 10.1038/srep37839 (PMC5122892; doi:10.1038/srep37839)
Supplement: Supplementary Data [file srep37839-s1.pdf]

A holistic approach to dissecting SPARC family protein complexity reveals FSTL-1 as an inhibitor of pancreatic cancer cell growth.

Katrina Viloria, Amanda Munasinghe, Sharan Asher, Roberto Bogyere, Lucy Jones and Natasha J Hill

Supplementary Table 1

Protein	Endothelial cells	Fibroblasts	Pancreatic stellate cells	β cells	β cells
	(HUVEC)	(MRC5)	(PS1)	(INS1)	(MIN6)
**Hevin 75 kDa**			110	110	110
	49	49	49	49	49
				39	
**SPOCK-1 49 kDa**			100		100
			56	56	56
	49	49	49	49	49
**SPOCK-2 47 kDa**			120	120	120
			60	60	60
	48	48			
**SPOCK-3 49 kDa**	65	65	65		
	53	53	53	53	53
	34	34	34		
**SMOC-1 48 kDa**	65				
			53	53	53
**SMOC-2 50 kDa**	nd	nd	nd	nd	nd
**FSTL-1 35 kDa**	40	40	40		

Supplementary Table 1: SPARC family isoforms and their expression in specific pancreatic cell types. Molecular weight quantification using Li-Cor Image Studio was performed for all isoforms observed in the western blot experiments shown in Figure 3. The table shows the typical band sizes (kDa) observed for each SPARC family protein in stromal cells (stellate cells, fibroblasts, endothelial cells) and β cells, taken from at least 2 independent blots. The predicted molecular weight for each SPARC family protein is indicated in the first column (the unmodified primary variant). nd = not detected.

Supplementary Table 2

	Predicted Glycosylation sites			Predicted Phosphorylation sites	
	**N-linked**	**O-linked**	**Total Glycosylation sites**	**Phosphorylation**	**Total Phosphorylation sites**
**Hevin**	Asn169, Asn176, Asn196, Asn280, Asn412, Asn476	Thr31, Thr40, Ser44, Thr116, Thr331, Thr398,	12	Ser92, Ser127, Thr149, Ser182, Ser414, Thr419, Ser420, Ser421	8
**SPOCK-1**		Ser131, Thr228, Ser383 (GAG), Ser388 (GAG)	4	Tyr65, Ser131, Ser144, Ser150, Thr346, Ser351, Thr352	7
**SPOCK-2**	Asn225	Ser383 (GAG), Ser388 (GAG)	3	Thr154, Tyr155, Ser156	3
**SPOCK-3**		Ser384 (GAG), Ser389 (GAG)	2	Ser223, Ser225, Tyr365, Ser372	4
**SMOC-1**	Asn214, Asn374	Ser37, Thr163, Ser168, Ser172, Thr300, Thr301, Ser351,	9	Thr155	1
**SMOC-2**	Asn206, Asn362		2	Tyr193	1
**FSTL-1**	Asn144, Asn175, Asn180		3	Ser165, Ser166, Thr279, Thr284, Tyr286	5

Supplementary Table 2: Predicted post-translational modifications of SPARC family proteins. Potential glycosylation and phosphorylation sites for each of the extended family of SPARC proteins were predicted using Genecards, UNIPROT, and Phosphosite Plus databases. Positions of known glycosaminoglycan (GAG) chain linkages are also indicated.

Supplementary Table 3

Gene name (total no. coding variants)	Transcript Name	ENSEMBL Transcript ID	Base pairs	Amino acids	Predicted Protein size (kDa)
SPARCL1 (Hevin) (10)	201	418378	2994	664	75
	001	282470	2906	664	75
	005	503414	2520	539	62
SPOCK-1 (5)	001	394945	4846	439	49
	201	282223	4488	377	33
SPOCK-2 (4)	004	373109	5445	424	47
	201	317376	5328	424	47
	203	536168	1824	423	47
	202	412663	1284	77	8
SPOCK-3 (17)	001	357154	2986	436	49
	002	502330	2180	436	49
	006	357545	2936	433	49
	012	511269	1768	433	49
	014	506886	2947	436	49
	015	511531	2908	436	49
	016	504953	2900	433	49
	013	510741	1963	393	44
	201	421836	3061	385	44
	017	535728	2007	344	39
	010	512681	1457	338	38
	202	541354	2797	316	37
	005	512648	1456	313	36
SMOC-1 (2)	001	361956	2040	435	48
	002	381280	3666	434	48
SMOC-2 (4)	002	354536	3150	457	51
	001	356284	3117	446	50
	201	535039	1875	136	14
FSTL-1 (4)	001	295633	5943	308	35
	004	424703	1396	273	31

Supplementary Table 3: Bioinformatics analysis of splice variants of SPARC family proteins. Alternative transcripts of the wider SPARC family were obtained from the ENSEMBL database. The total number of CDS transcripts is indicated in the first column. Only protein coding variants for which the complete CDS is known were included in further analyses. For these sequences, FASTA sequences were downloaded and the respective product molecular weight was calculated using the Protein Molecular Weight Bioinformatics tool.

Supplementary Table 4

Primer name	sequence	primary target transcript ID	primary PCR prod length	Transcripts with differential product length
**GenAFor**	TCGGACGGCGGTAATTTTCT	006 (generic)	158	201 (238 bp), long variants with exon 8/9 (167 bp)
**GenARev**	GGATCTAAAGCCTGATCGAAGG			
**GenBFor**	CGCACTTGGAGTCCAGGAAA	006 (generic)	740	202 (625 bp), 013 (620 bp), 018 (386 bp)
**GenBRev**	CAAGGTGGGTCTTGCTGTCT			
**005For**	AGAAATGTTAAGAGAGCATGCAG	005	386	
**005Rev**	TTGCGTCTGTAAGGGTCTCA			
**202Rev**	CCTGCTTCTTTCATCCCTGATCG	202	154	
**010For**	TTCCGAGACTGCAAACTAGAAT	010	476	
**013Rev**	GCTGGTATCGAATCCTCTCTTAAC	013	402	
**201For**	GATCACACAAGATCACATCCACA	201	215	
**201Rev**	CTTCTTTCATCCTGTGTGTAAGCC			

Supplementary Table 4: SPOCK-3 primers used for splice variant analysis.

Supplementary Table 5

	Immunogen	Stock Concentration	IHC titrations	Optimal IHC concentration	WB concentration	Manufacturer
**SPARCL1/ Hevin**	215-244	0.5 mg/ml	1/100-1/800	1/100-1/200	1/300	Abcam (ab107533)
**SPARCL1/ Hevin**	649-664 C terminus	0.5 mg/ml			1/500	LS Bio (LS-C313206)
**SPOCK-1**	31-61	0.25 mg/ml	1/25 - 1/200	1/25-1/50	1/500	Abcam (ab174479)
**SPOCK-2**	200-250	0.4 mg/ml	1/100 - 1/400	1/100-1/200	1/300	Novus Bio (NBPI-92442)
**SPOCK-3**	71-365	1 mg/ml	1/200 - 1/800	1/200-1/400	1/500	Abcam (ab111897)
**SMOC-1**	140-363	0.9 mg/ml	1/50 - 1/200	1/100-1/200	1/500	Abcam (ab155776)
**SMOC-2**	N terminus	0.5 mg/ml	1/25 - 1/400	1/100-1/200	1/500	Abcam (ab78069)
**FSTL-1**	Internal	0.3 mg/ml	1/50 - 1/100	1/50	1/300	Novus Bio (NBPI-83425)
**SPARC**	1-90	200 µg/ml			1/1000	Santa Cruz (H-90)

Supplementary Table 5: SPARC family antibodies used in the study. Table shows details of the SPARC family antibodies used, including manufacturer and concentrations used.

Supplementary Figure 1

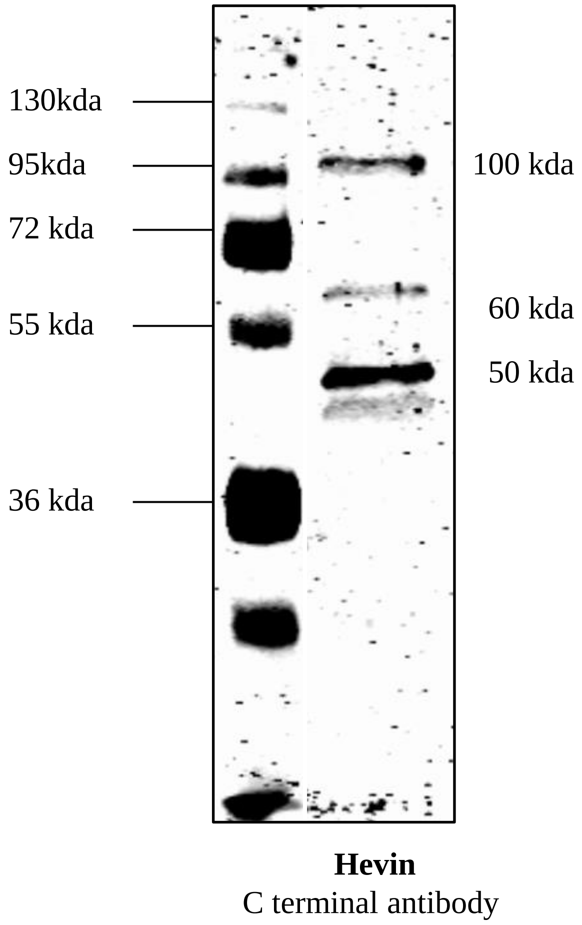

Supplementary Figure 1: Detection of Hevin isoforms using a C-terminal antibody. Isoforms of Hevin expressed in PS-1 cells were determined by western blot using an anti-Hevin antibody specific to the C' terminus of Hevin. Images representative of n=3 from 3 independent replicates, where the labelled bands were consistently observed.

Supplementary Figure 2

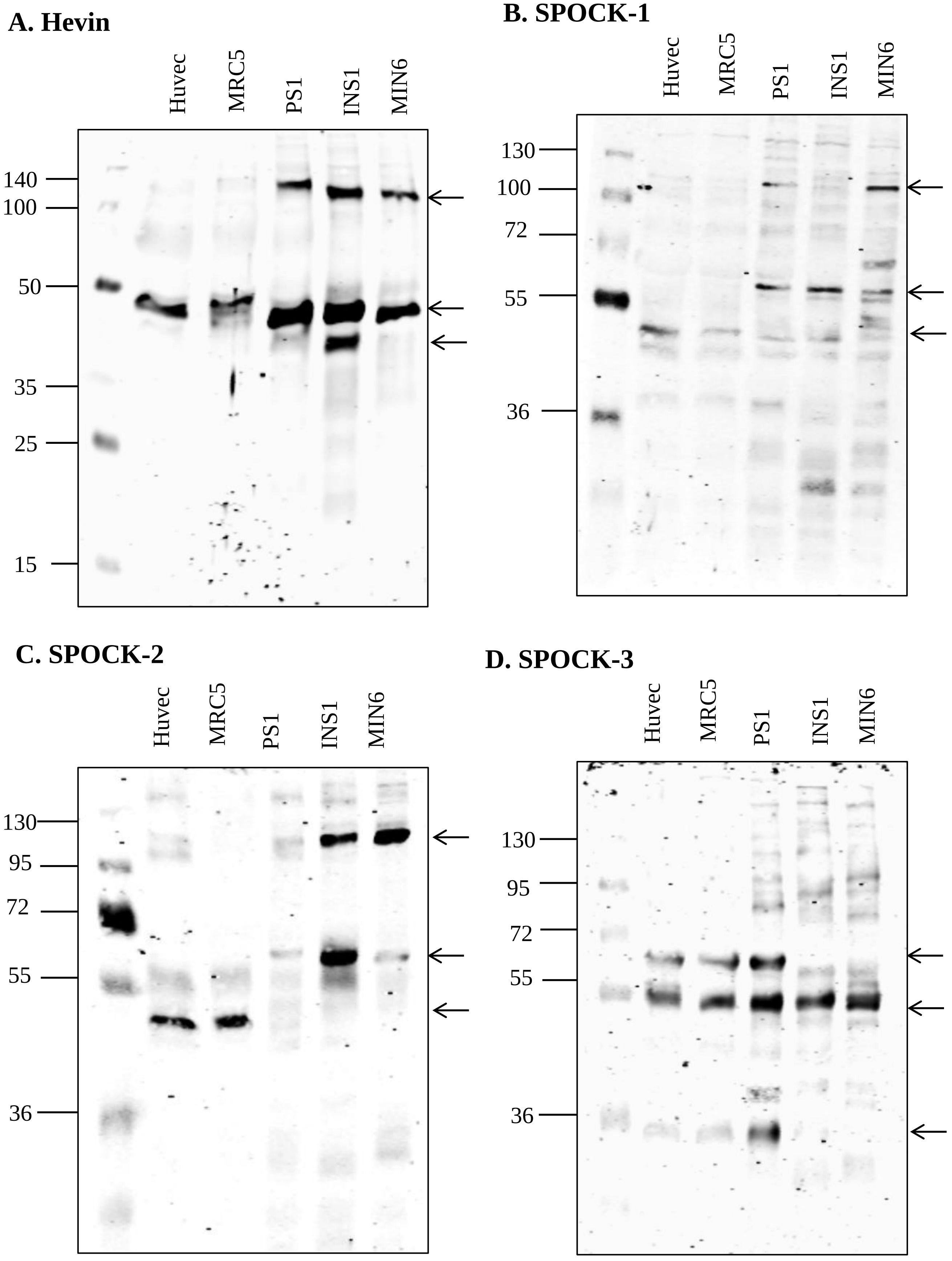

Supplementary Figure 2

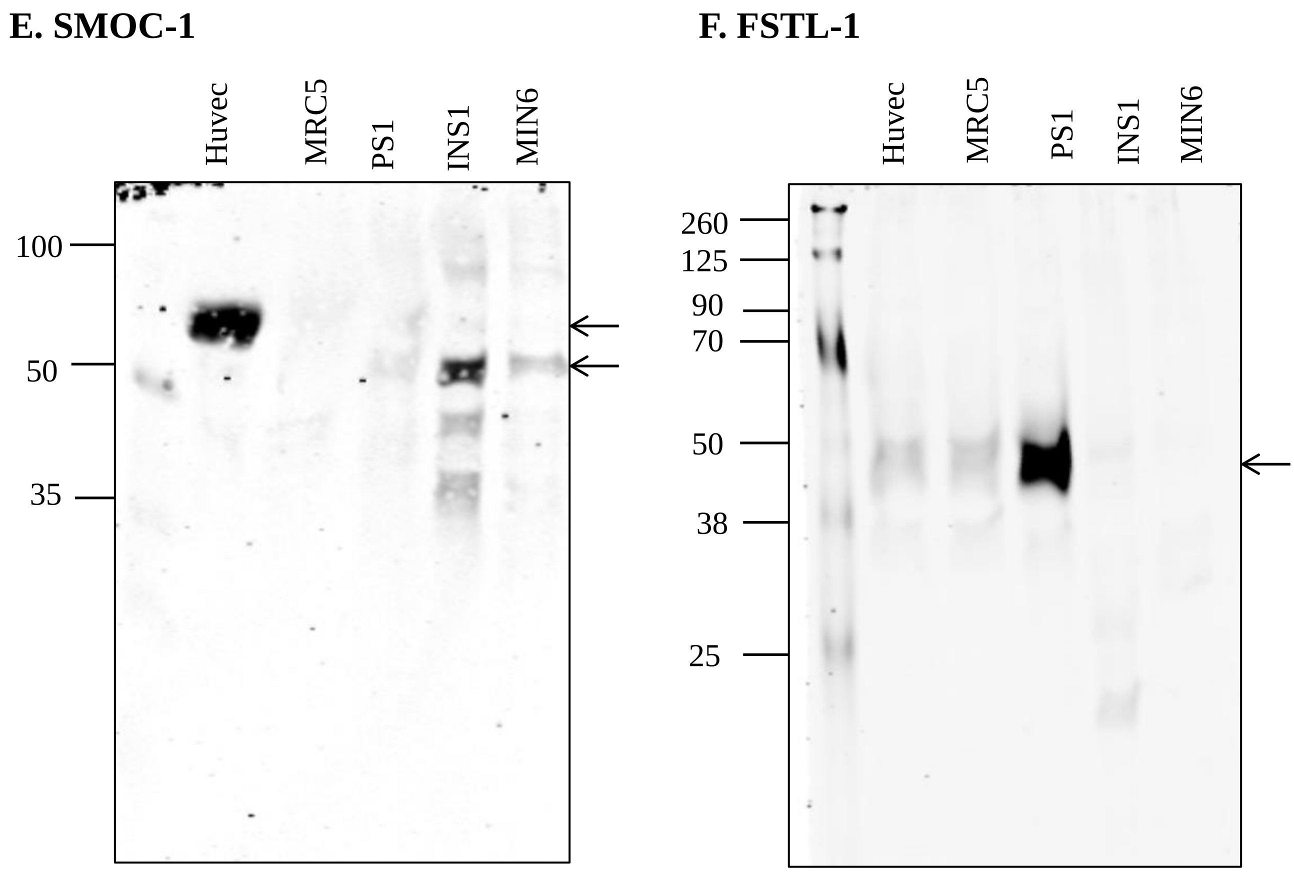

Supplementary Figure 2: Full blots for cropped images shown in Figure 5. Arrows indicate bands observed across multiple experiments that are shown in Figure 5.

Supplementary Figure 3

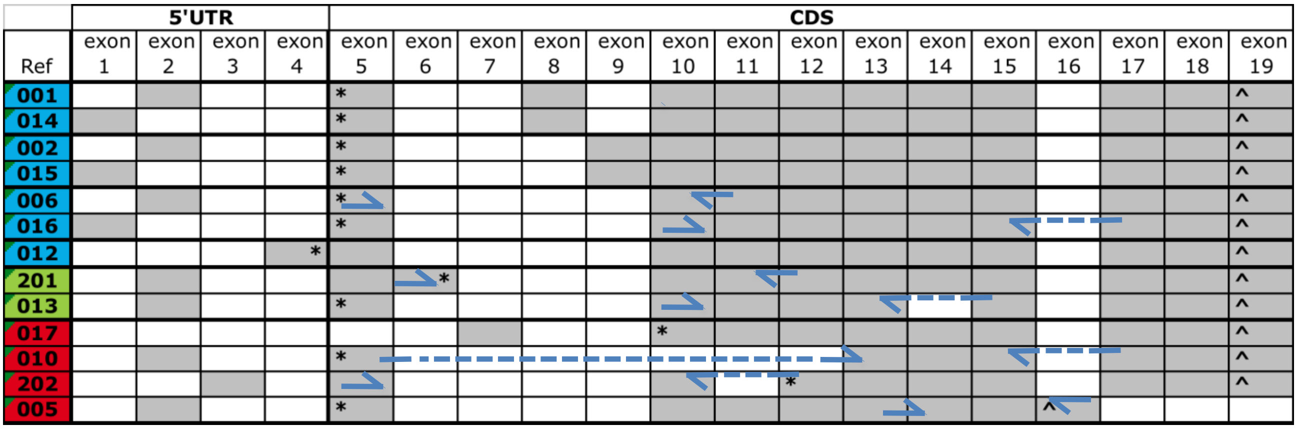

Supplementary Figure 3: Exon structure of SPOCK-3 splice variants. Splice variants banked in ENSEMBL (Accessed March 2013) for SPOCK-3 (ENSG 196104), showing 5'UTR and CDS exons. The first column indicates the ENSEMBL transcript reference. Shaded boxes indicate exons present in each variant. Long variants are designated in blue (49 kDa), medium in green (44 kDa), small in red (36-39 kDa). Shading indicates the presence of the particular exon. * indicates translational start site, while ^ indicates translation termination site. Diagram is not to scale. Blue arrows indicate position of forward and reverse primers. The primers shown on transcript 006 and 0016 indicate position of GenA and GenB primers, respectively. All other primers correspond to the indicated transcript. Primer sequences and predicted PCR product sizes are given in Supplementary Table 2.

Supplementary Figure 4

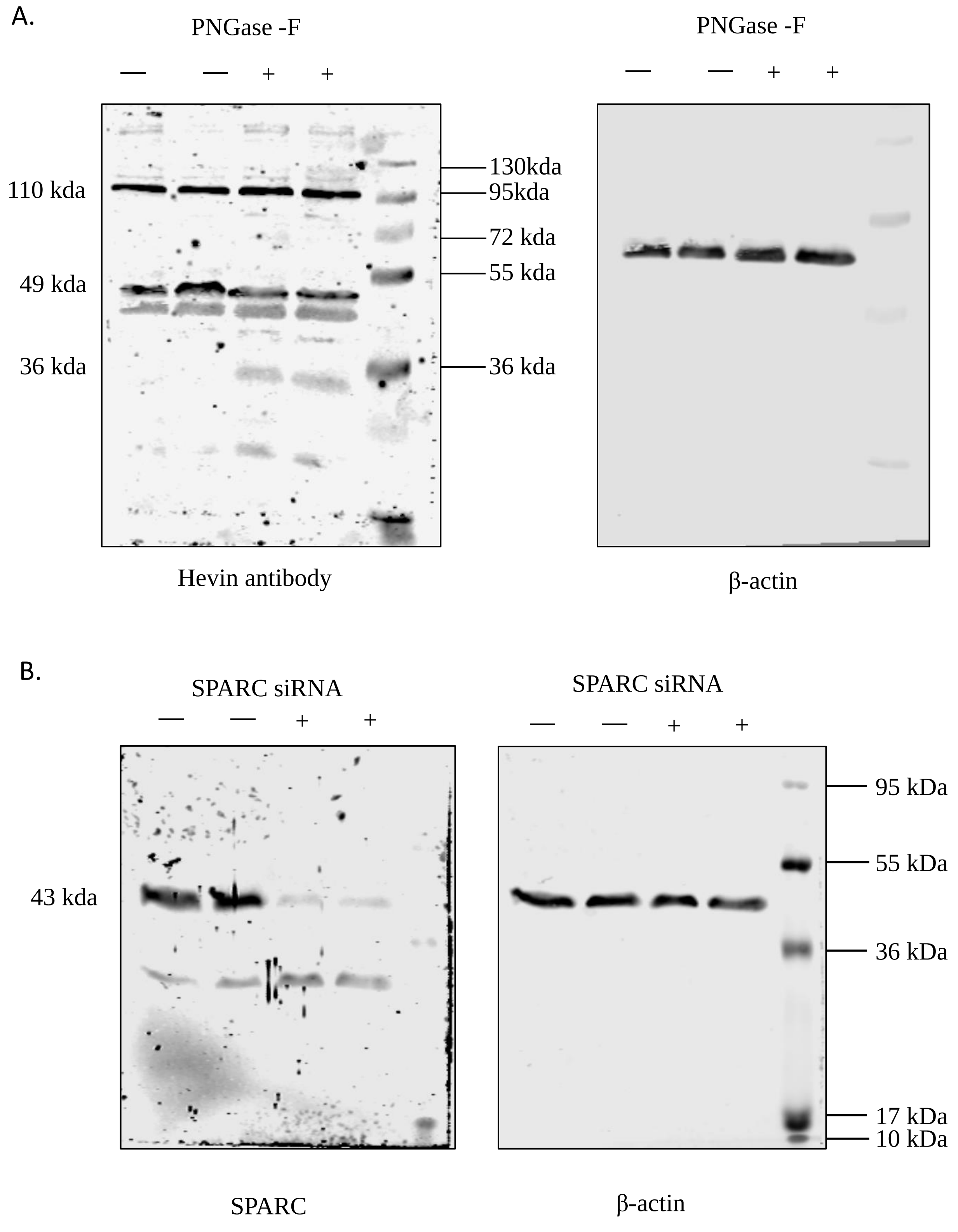

Supplementary Figure 4

C.

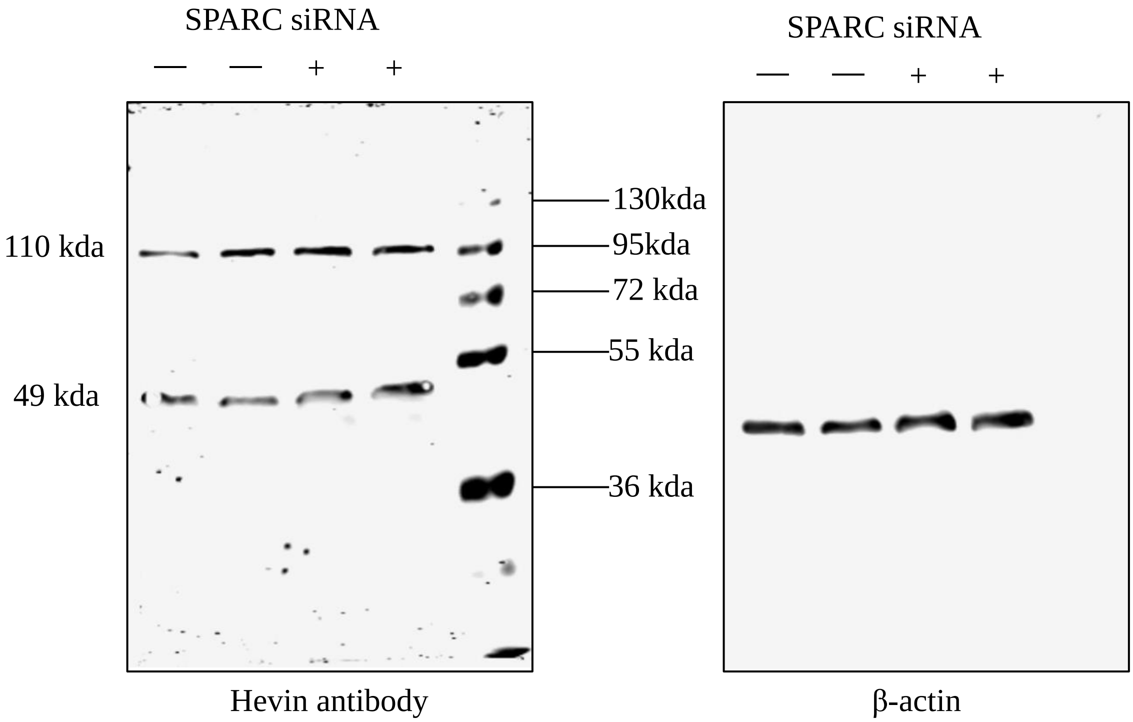

D.

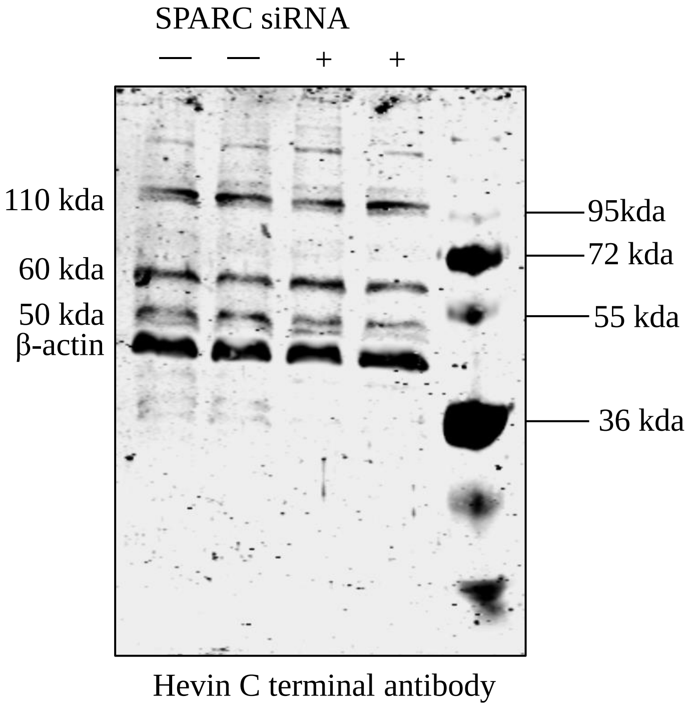

Supplementary Figure 4: Full blots for cropped images in Figure 7. See Figure 7 legend for experimental details. In (B), the ~30 kDa band detected with the SPARC antibody is a routinely observed non-specific band that is not affected by SPARC siRNA treatment.

Supplementary Figure 5

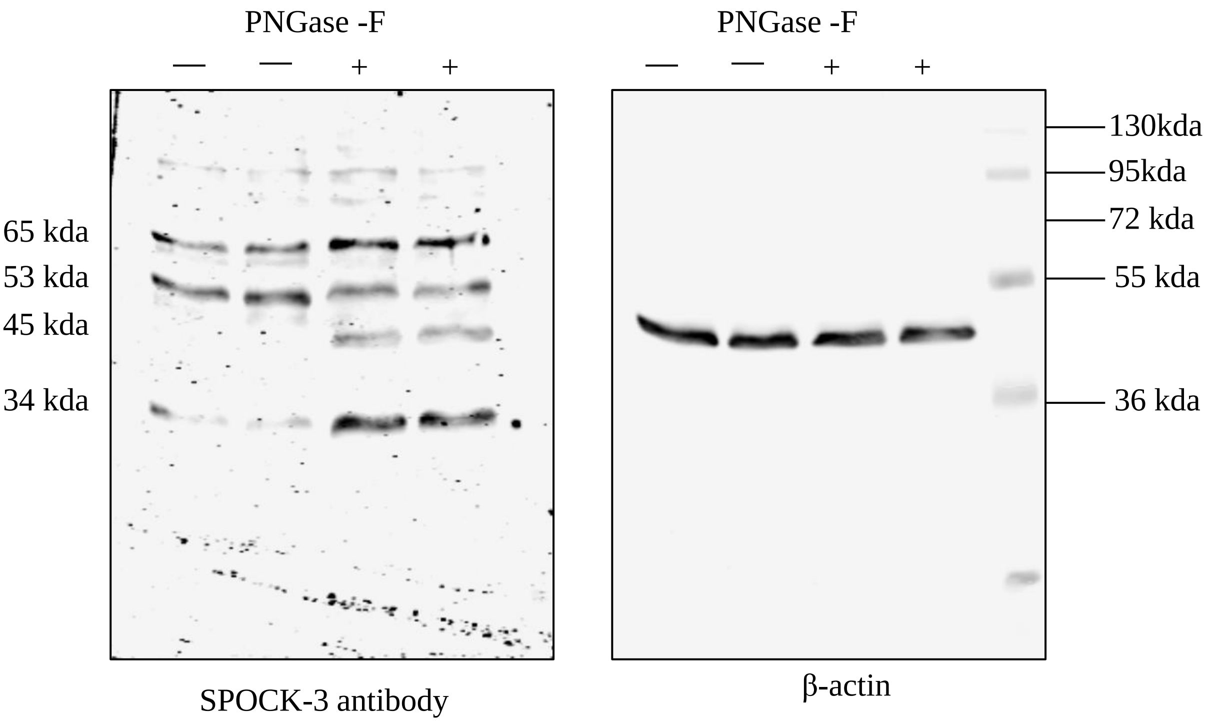

Supplementary Figure 5: Full blots for cropped images in Figure 8. See Figure 8 legend for experimental details.

Supplementary Figure 6

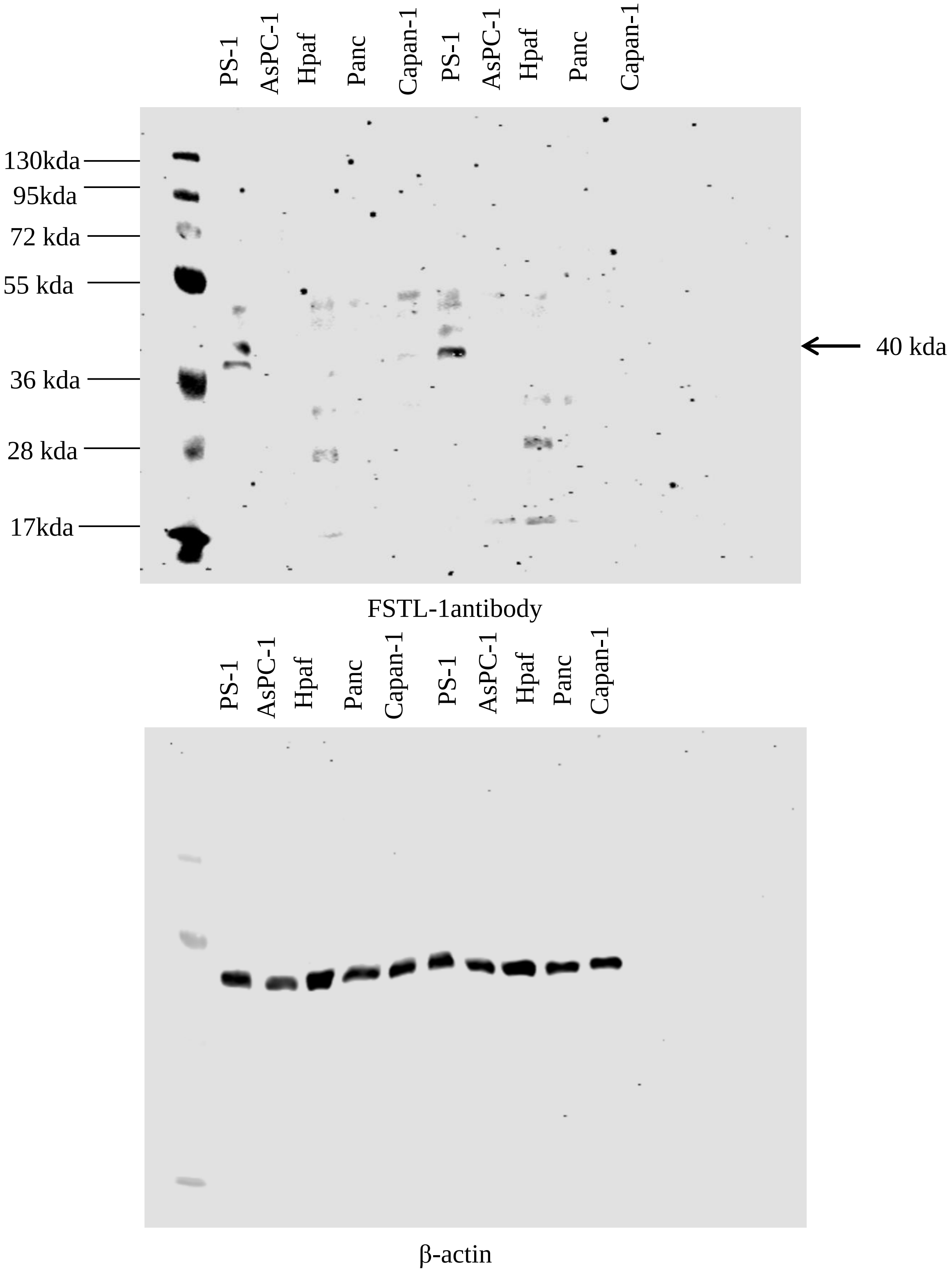

Supplementary Figure 6: Full blots for cropped blot in Figure 10D. See Figure 10 legend for experimental details. Arrow indicates consistently observed band at 40 kDa for FSTL-1.
